# Procalcitonin-guided antibiotic therapy in ICU patients with sepsis: a systematic review and meta-analysis with GRADE and trial sequential analysis

**DOI:** 10.62838/jccm-2026-0043

**Published:** 2026-10-30

**Authors:** Hanan Abdullah Alharthi, Ehab Abdulrahman Abdu, Abeer Adel Alshaer, Layan Adel Alshaer, Mansour Abdullah Alghamdi, Yara Al-Dosari, Amani Abualnaja, Ruba Abdulrahman Almubarriz, Rahaf Ahmed Alamer, Maha Ali Bakeet Alsqoor, Amjad Falah Saeed Alshahrani, Amjad Mohammed Ali Majrashi, Shooq Mnbat Al-Shahrani, Alhassan Rizq Alqarih, Reem Hamzah Alshelali

**Affiliations:** Internal Medicine Department, King Abdulaziz Hospital, Jeddah, Saudi Arabia; Internal Medicine Department, Faculty of Medicine and Health Sciences, Sana'a University, Sana'a, Yemen; Internal Medicine Department, Bisha University, Bisha, Saudi Arabia; College of Applied Medical Sciences, University of Bisha, Bisha, Saudi Arabia; Internal Medicine Department, King Abdullah Bin Abdulaziz University Hospital, Riyadh, Saudi Arabia; Internal Medicine Department, King Faisal Specialty Hospital & Research Centre, Riyadh, Saudi Arabia; General Surgery Department, Riyadh First Health Cluster, Riyadh, Saudi Arabia; Internal Medicine Department, Najran University, Najran, Saudi Arabia; Internal medicine Department, King Abdullah Medical Complex, Jeddah, Saudi Arabia

**Keywords:** sepsis, procalcitonin, antibiotic stewardship, mortality, intensive care unit (ICU)

## Abstract

**Objective:**

To evaluate the efficacy and safety of PCT-guided antibiotic therapy compared with standard care in adult intensive care unit (ICU) patients with sepsis or septic shock.

**Methods:**

PubMed, Scopus, the Cochrane Central Register of Controlled Trials (CENTRAL), and Web of Science were searched from database inception to January 2026 to identify randomized controlled trials (RCTs) comparing PCT-guided antibiotic strategies with standard care in adult ICU patients with suspected or confirmed bacterial infections. The primary clinical outcome was all-cause mortality at the longest available follow-up, whereas the key antimicrobial stewardship outcome was duration of antibiotic therapy. Secondary outcomes included infection relapse, new infection or superinfection, antimicrobial-free days, clinical cure, and length of ICU or hospital stay. Random-effects meta-analyses were performed using intention-to-treat data. Trial sequential analysis (TSA) and the GRADE framework were applied to assess the conclusiveness and certainty of the evidence.

**Results:**

Sixteen RCTs involving 7,966 patients were included. PCT-guided therapy did not reduce all-cause mortality compared with standard care (RR = 0.99, 95% CI 0.92 to 1.06; p = 0.72), with trial sequential analysis indicating insufficient evidence to draw a definitive conclusion. In contrast, PCT-guided strategies significantly reduced antibiotic duration (MD = −1.51 days, 95% CI −2.24 to −0.78), with trial sequential analysis confirming a conclusive benefit. Procalcitonin guidance was associated with an increase in antimicrobial-free days without higher rates of new infection, superinfection, or clinical failure. Additionally, no significant differences were observed in ICU or hospital length of stay.

**Conclusions:**

In adult ICU patients with sepsis or septic shock, RCT-guided antibiotic strategies were associated with reduced antibiotic exposure and increased antibiotic-free days, without a detectable improvement in survival. These findings support the use of procalcitonin as an adjunctive antimicrobial stewardship tool, provided that it is integrated with clinical assessment, microbiological data, and sound clinical judgment.

## Introduction

Sepsis is a life-threatening condition characterized by infection-induced immune dysregulation, resulting in acute organ dysfunction and high mortality among critically ill patients worldwide [1,2]. The timely initiation of appropriate antimicrobial therapy is a cornerstone of international sepsis management and is consistently associated with improved survival and clinical outcomes [3,4]. However, effective antimicrobial management also requires careful determination of the optimal timing for antibiotic de-escalation or discontinuation to minimize antimicrobial resistance, drug-related adverse events, secondary infections, and avoidable healthcare costs [5,6,7,8]. In this context, relying solely on clinical improvement and nonspecific inflammatory markers may delay antibiotic discontinuation, especially in critically ill patients with ongoing organ dysfunction. Therefore, adjunctive tools indicating resolution of infection may assist clinicians in making individualized decisions regarding the duration of antibiotic therapy.

Several biomarkers have been described for assessing infection severity and guiding antibiotic de-escalation strategies, with leukocyte count and C-reactive protein (CRP) being the most commonly used in clinical practice [9]. However, these markers may lack sufficient specificity in critically ill patients because they can be influenced by trauma, surgery, systemic inflammation, and noninfectious organ dysfunction. Among recent available biomarkers, procalcitonin, a calcitonin-related gene product expressed by human epithelial cells in response to bacterial infections, has gained more attention as a biomarker with superior specificity and sensitivity compared with CRP for monitoring the course of severe bacterial infections [10,11,12,13]. In February 2017, the US Food and Drug Administration approved procalcitonin as a blood biomarker to support guidance of antibiotic therapy in patients with sepsis. Current sepsis guidance suggests that procalcitonin may be considered as an adjunct to support antibiotic discontinuation decisions in selected critically ill patients, while emphasizing that its use should complement rather than replace clinical judgment [14]. Although most randomized trials of procalcitonin-guided strategies were designed to evaluate reductions in antibiotic exposure, emerging evidence suggests that these interventions may also influence mortality outcomes in critically ill populations. In this regard, a large, randomized trial conducted in the Netherlands reported a reduction in mortality associated with PCT-guided antibiotic therapy [5], whereas the recent ADAPT-Sepsis trial demonstrated a non-significant difference in all-cause mortality at the last time point compared with standard care [15]. Given the conflicting results and a lack of robust evidence regarding the consistency of potential benefits across different mortality time points, sepsis severity strata. Therefore, a comprehensive meta-analysis of RCTs is warranted to clarify the efficacy and safety of PCT-guided antibiotic therapy compared with standard care in adult patients with sepsis or septic shock and to inform evidence-based clinical practice recommendations.

## Methods

### Protocol Registration and Reporting Standards

This systematic review and meta-analysis protocol was developed in accordance with the Preferred Reporting Items for Systematic Reviews and Meta-Analyses (PRISMA) [16] and follows the methodological guidance outlined in the Cochrane Handbook for Systematic Reviews of Interventions [17]. The protocol was registered in PROSPERO under registration number (CRD420261410435).

### Information Sources and Search Strategy

A comprehensive and systematic literature search was conducted in MEDLINE (via PubMed), Scopus, the Cochrane Central Register of Controlled Trials (CENTRAL), and Web of Science (WoS) from database inception to January 2026, to identify RCTs evaluating PCT-guided antibiotic therapy compared with standard care or usual care in critically ill patients admitted to the intensive care unit (ICU) with sepsis, septic shock, or suspected bacterial infection. A highly sensitive search strategy was developed using Boolean operators and a combination of MeSH terms and free-text keywords, including: “procalcitonin,” “PCT-guided therapy,” “antibiotic discontinuation,” “standard care,” “sepsis,” “septic shock,” “bacterial infection,” and “intensive care unit”. No restrictions were applied regarding language, publication status, or publication date. A detailed search strategy for each database, including all search terms, Boolean operators, and filters, is provided in the Supplementary Table 1. To ensure comprehensive coverage, forward and backward citation tracking was performed by manually screening the reference lists of included studies and relevant systematic reviews, as well as identifying newer studies citing the included articles using Google Scholar. In addition, ClinicalTrials.gov and the WHO International Clinical Trials Registry Platform (ICTRP) were searched to identify ongoing or unpublished trials.

### Study Selection

All retrieved records were initially imported into EndNote reference management software for duplicate removal. The deduplicated records were subsequently uploaded to the Rayyan web-based screening tool for further evaluation [18]. Study selection was conducted in two sequential stages. First, titles and abstracts were independently screened by two reviewers to assess eligibility according to the predefined inclusion and exclusion criteria. Second, the full texts of potentially eligible studies were retrieved and independently assessed for final inclusion. Any discrepancies between reviewers at either stage were resolved through discussion, and when consensus could not be reached, a third reviewer was consulted.

### Eligibility Criteria and Outcomes

We included randomized controlled trials (RCTs) enrolling adult ICU patients with suspected or confirmed bacterial infection, including suspected sepsis, sepsis, severe sepsis, or septic shock, regardless of infection source, as defined by the original trial investigators using SIRS-based criteria, Sepsis-2, Sepsis-3, or trial-specific definitions of suspected infection. Eligible interventions included PCT-guided antibiotic strategies in which initiation, continuation, escalation, de-escalation, or discontinuation of systemic antibiotic therapy was guided by serial procalcitonin measurements according to a predefined algorithm or protocol. Comparator groups consisted of standard care, usual care, or guideline-based antibiotic management relying on clinical judgment without reference to procalcitonin values. Only RCTs conducted in ICU settings were included, with no restrictions on language or publication date. Studies were excluded if they involved pediatric populations, were conducted exclusively outside the ICU, evaluated biomarkers other than procalcitonin without a PCT-guided algorithm, used non-randomized designs, or focused on infections requiring predefined prolonged antibiotic therapy where biomarker-guided discontinuation was not clinically appropriate. Eligible studies were required to report at least one prespecified outcome of interest. The primary clinical outcome was all-cause mortality at the longest available follow-up, whereas the key antimicrobial stewardship outcome was duration of antibiotic therapy. The secondary outcomes included length of ICU stay or hospital stay, infection relapse or recurrence, new infection or superinfection, antibiotic-free days, and clinical cure.

### Data Extraction

Data extraction was performed using a predefined, structured data extraction sheet developed in Microsoft Excel. The extraction sheet captured key information across three main domains: study summary characteristics, baseline patient characteristics, and outcome data (including all prespecified primary and secondary outcomes). Data were extracted independently by two reviewers. Any discrepancies identified during the extraction process were resolved through discussion and consensus, and when agreement could not be achieved, a third reviewer was consulted.

### Risk of Bias Assessment

The risk of bias of included RCTs was independently assessed by two reviewers using the Cochrane Risk of Bias 2 (RoB 2) tool [19], which evaluates bias arising from the randomization process, deviations from intended interventions, missing outcome data, measurement of outcomes, and selection of the reported results. Each study was judged as having low risk of bias, some concerns, or high risk of bias according to the RoB 2 algorithm. Any disagreements between reviewers were resolved through discussion and consensus.

### Data synthesis and statistical analysis

All statistical analyses were performed using Stata/SE version 19.5 (StataCorp, College Station, TX). Data from all eligible trials were analyzed strictly according to the intention-to-treat (ITT) principle, whereby participants were retained in their originally randomized groups regardless of treatment adherence, crossover, or protocol deviations. For dichotomous outcomes, treatment effects were pooled as risk ratios (RRs) with corresponding 95% confidence intervals (CIs). For continuous outcomes, effects were summarized as mean differences (MDs) with 95% CIs. When continuous data were reported as medians with interquartile ranges or ranges, these were converted to means and standard deviations using established statistical methods before pooling. Study-specific effect sizes were pooled using a random-effects DerSimonian–Laird model, accounting for anticipated clinical and methodological heterogeneity across studies. Statistical heterogeneity was assessed using Cochran's Q test, the I^2^ statistic, and between-study variance (τ^2^), with I^2^ values of approximately 25%, 50%, and 75% interpreted as indicating low, moderate, and substantial heterogeneity, respectively. Additional graphical and exploratory analyses were performed to assess consistency and potential biases. L'Abbé plots were generated to visualize the risk–benefit ratio for each included study, while Galbraith plots were used to identify potential outliers and sources of heterogeneity. Funnel plots were constructed to visually assess small-study effects and publication bias when a sufficient number of studies were available. To assess the robustness of the accumulated evidence and to minimize the risk of random errors arising from repeated significance testing, Trial Sequential Analysis (TSA) [20,21,22] was applied to both dichotomous and continuous outcomes. For dichotomous outcomes, we prespecified a two-sided α of 0.05, β of 0.20 (80% power), and an anticipated relative risk reduction of 10%, with the control event rate estimated from the pooled control groups across included trials. For continuous outcomes, the required information size was calculated using the observed variance, a prespecified clinically meaningful mean difference, and adjustment for between-study heterogeneity based on the random-effects model variance. In both analyses, O'Brien–Fleming α- and β-spending boundaries were applied. The evidence was considered conclusive if the cumulative Z-curve crossed the conventional significance boundary and the trial sequential monitoring boundary, or if the required information size was reached; otherwise, the evidence was considered inconclusive, indicating that further studies may be necessary.

The Grading of Recommendations Assessment, Development, and Evaluation (GRADE) [23] framework was used to assess the certainty of the evidence and the strength of recommendations. Certainty of evidence was classified as high, moderate, low, or very low. High certainty indicates strong confidence that the estimated effect is close to the true effect, whereas moderate certainty suggests that further research may affect the estimate. Low certainty implies that additional studies are likely to substantially influence the effect estimate, and very low certainty reflects considerable uncertainty, with the true effect potentially differing markedly from the estimated effect. The GRADE approach provides a transparent and systematic method for translating evidence into clinical recommendations.

Prespecified subgroup analyses were conducted for mortality according to follow-up time points (ICU, in-hospital, 28-day, 60-day, and 90-day mortality), and for both all-cause mortality and duration of antibiotic therapy according to baseline illness severity stratified by SOFA score categories (0–6, 7–9, and ≥10).

## Results

### Literature Search

The literature search identified a total of 6,730 records from electronic databases. After removal of 687 duplicate records, 6,043 records remained for title and abstract screening, of which 5,950 were excluded as clearly irrelevant. Consequently, 93 full-text articles were retrieved and assessed in detail for eligibility. Following full-text review, 16 RCTs met the eligibility criteria and were included in both the systematic review and meta-analysis [1,5,15,24,25,26,27,28,29,30,31,32,33,34,35,36]. The study selection process is illustrated in the PRISMA flow diagram (Figure 1).

**Fig. 1.**
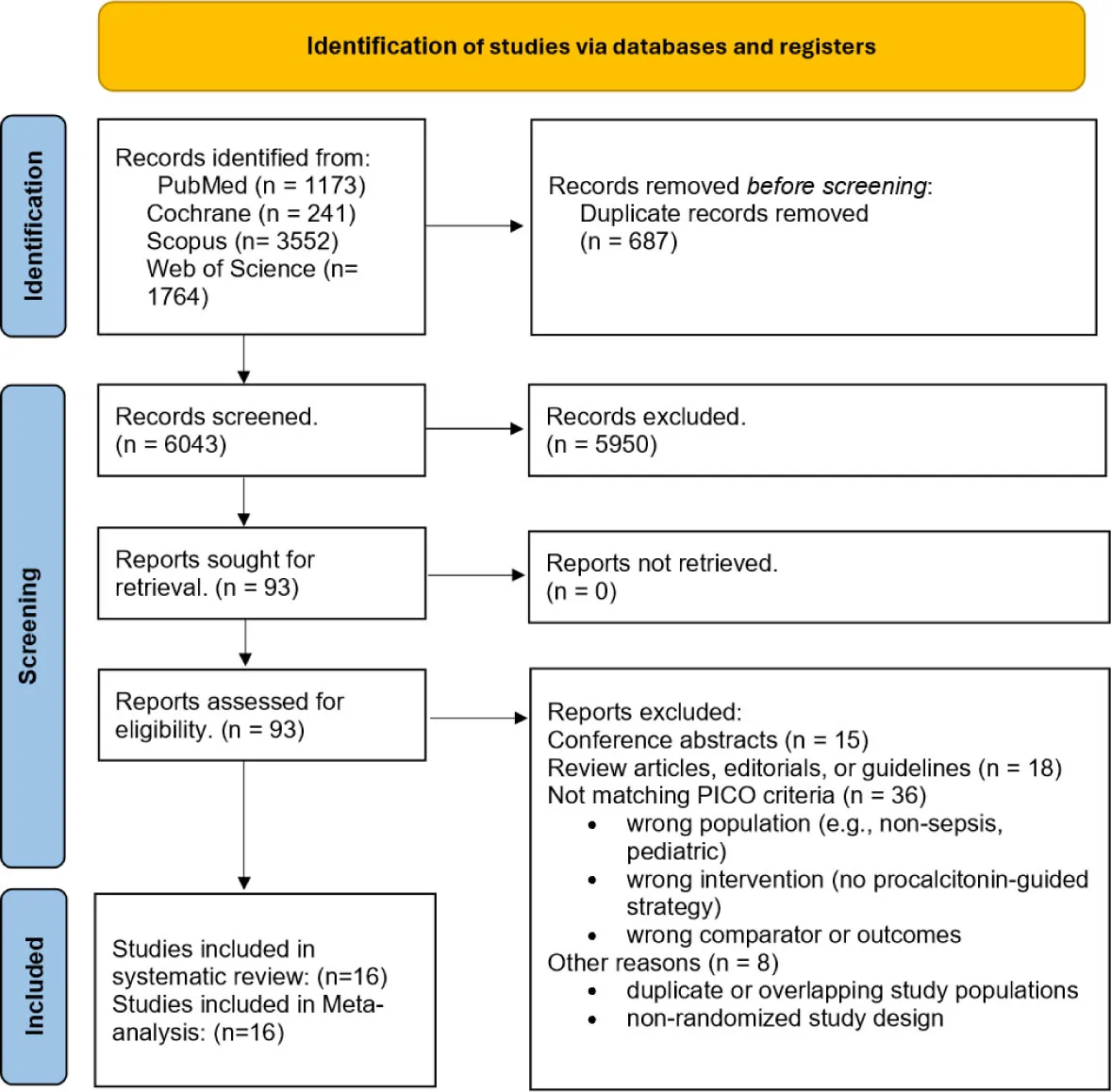
PRISMA flow diagram

### Characteristics of the included studies and risk of bias assessment

Sixteen RCTs enrolling 7,966 patients were included, of whom 3,985 were allocated to a PCT-guided antibiotic strategy and 3,981 to standard care. Across trials, baseline demographic characteristics were comparable between groups. The overall weighted mean age of enrolled patients was 63.5 years, and 4,747 patients (59.6%) were male. Follow-up duration ranged from 28 to 365 days, with a median follow-up of 60 days and a mean follow-up of 93.4 days. A detailed summary of study characteristics and baseline demographics is presented in Table 1. Risk of bias assessment using the Cochrane RoB-2 tool demonstrated that the majority of included randomized trials were judged to be at low overall risk of bias or to raise some concerns. In contrast, Hochreiter et al. (2009) [28] was assessed as being at high risk of bias, primarily due to insufficient reporting of the randomization process and lack of adequate allocation concealment (Figure 2).

**Fig. 2.**
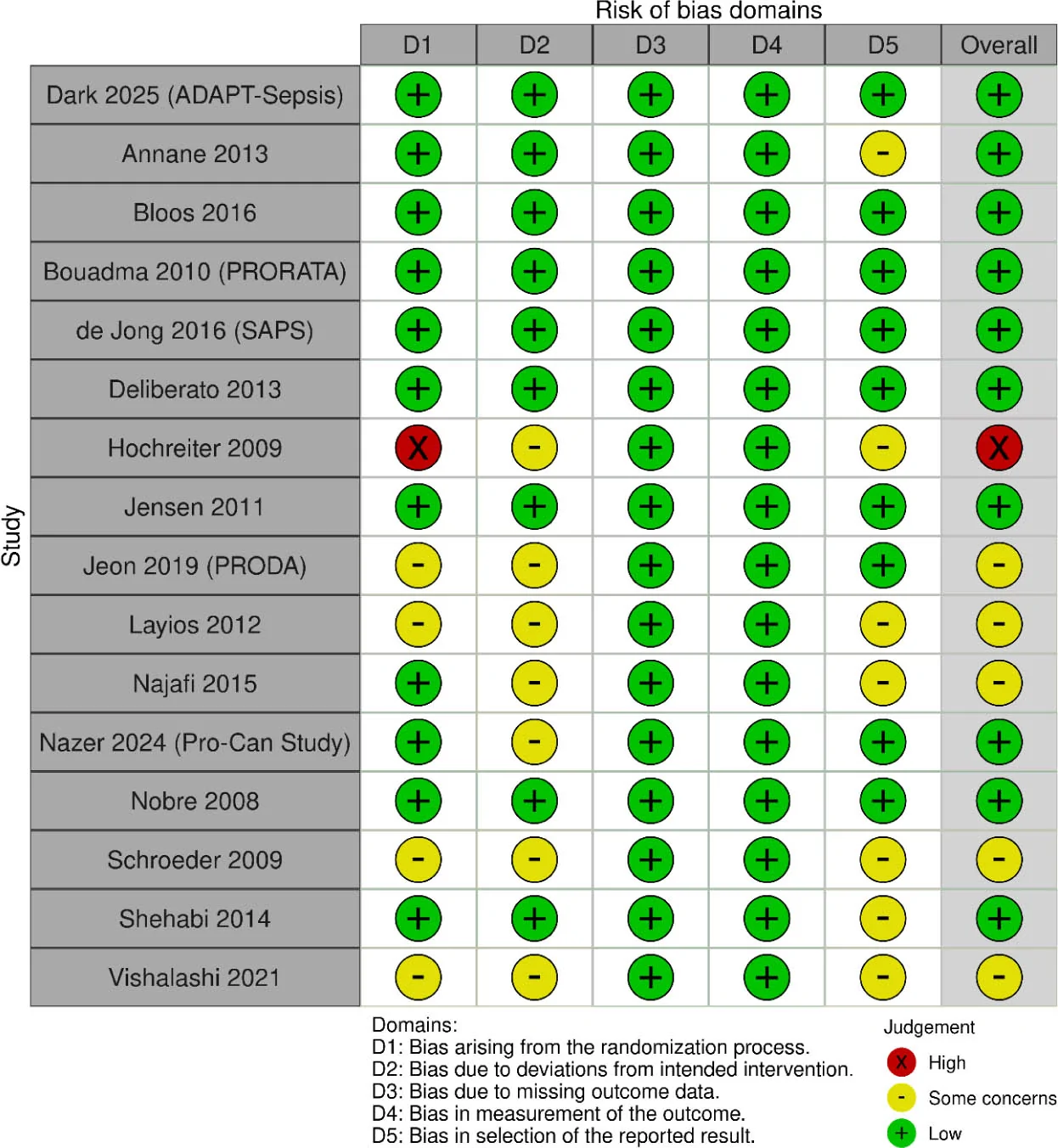
Risk of bias assessment (ROB-2) for the included studies.

**Table 1:** Baseline characteristics of patients included in this review.

**Study ID**	**Study design**	**Setting, type of trial**	**Country**	**Time frame**	**Follow-up (days)**	**Total**	**Sample size in ITT analysis PCT-guided**	**Control**	**Clinical diagnosis**	**Definition of sepsis**	**Type of procalcitonin algorithm and procalcitonin cutoffs used**	**Primary outcomes**
Dark 2025 (ADAPT-Sepsis)	Multicenter, intervention-concealed RCT	41 UK 41 UK National Health Service ICUs	UK	January 1, 2018, to June 5, 2024	90-days	1836	918	918	Suspected sepsis with acute organ dysfunction	Sepsis-3 (SOFA score)	Strong stop: PCT <0.25 μg/L; Supports stop: PCT drop >80% and <0.50 μg/L; Usual care otherwise	Total antibiotic duration (28d); 28-day all-cause mortality
Annane 2013	Multicenter, randomized, single-blind trial	8 ICUs in France	France	Dec 2006 to Dec 2009	Until hospital discharge or up to 30 days	62	31	31	Severe sepsis/septic shock	Severe sepsis phenotype, negative cultures, no overt infection	Algorithm at 6h, day 3, day 5: <0.25 μg/L (stop), 0.25–<0.5 (discourage), 0.5–<5 (recommend), ≥5 (strongly recommend)	Proportion on antibiotics at day 5
Bloos 2016	Multicenter, randomized, 2×2 factorial trial	33 ICUs in Germany	Germany	November 6, 2009, to March 8, 2013	90 days	1089	552	537	Severe sepsis or septic shock	Severe sepsis/septic shock (SIRS + organ dysfunction)	PCT measured on days 0,1,4,7,10,14; Stop if PCT ≤1 ng/mL or drop ≥50% from previous	28-day all-cause mortality
Bouadma 2010 (PRORATA)	Multicenter, prospective, parallel-group, open-label RCT	8 ICUs (medical/surgical), multicentre RCT	France	June 2007 to May 2008	60 days	630	311	319	Suspected bacterial infection in the ICU	Septic shock is defined per ACCP/SCCM criteria	Start: PCT <0.25→strongly discourage; >1→strongly encourage. Stop: PCT <80% of peak or <0.5 μg/L → encourage stop.	Mortality at 28 & 60 days (non-inferiority); days without antibiotics by day 28 (superiority)
de Jong 2016 (SAPS)	Multicenter, randomized, open-label intervention trial	15 ICUs (Netherlands), pragmatic RCT	Netherlands	Sept 18, 2009, to July 1, 2013	365 days	1575	776	799	Assumed or proven infection (sepsis, severe sepsis, or septic shock) in the ICU	Sepsis/severe sepsis/septic shock (2001 SCCM/ESICM criteria)	Stop if PCT ↓ ≥80% from peak or ≤0.5 μg/L. Non-binding advice.	Daily defined doses & duration of antibiotic treatment
Deliberato 2013	Single-centre, prospective, open-label RCT	1 ICU (Brazil)	Brazil	March 2008 to February 2010	Hospital discharge	81	42	39	Critically ill patients admitted with suspected sepsis, severe sepsis, or septic shock	Sepsis/Severe sepsis/Septic shock (ACCP/SCCM)	Stop if PCT ↓ >90% from peak or <0.5 ng/mL.	Duration of antibiotic therapy
Hochreiter 2009	Randomized prospective controlled trial	Surgical ICU, single center	Germany	January 2006 to March 2007	Until discharge	110	57	53	Suspected or confirmed bacterial infection + ≥2 SIRS criteria	ACCP/SCCM sepsis definition	Stop if clinical improvement + PCT <1 ng/mL or PCT >1 ng/mL but dropped to 25–35% of initial value over 3 days	Duration of antibiotic therapy
Jensen 2011	Randomized controlled open-label trial	9	Denmark	September 1, 2006, to February 6, 2009	60 days	1,200	604	596	Suspected infection in ICU patients	ACCP/SCCM criteria (sepsis, severe sepsis, septic shock)	Alert PCT: ≥1.0 ng/mL and not decreasing ≥10% from previous day → escalate antibiotics + intensify diagnostics	28-day all-cause mortality
Jeon 2019 (PRODA)	Multi-center randomized controlled trial	4 medical ICUs, single-blinded	Korea	July 2014 through June 2015	28 days	62	30	32	Suspected severe sepsis or septic shock, within 24h of ICU admission	Surviving Sepsis Campaign definitions	Stop if PCT decreased by ≥80% from peak or ≤0.5 μg/L; measured every other day	Duration of antibiotic therapy
Layios 2012	Randomized controlled trial (RCT)	Five ICUs from a tertiary teaching hospital	Belgium	April 2008 to December 2008	ICU stay	509	258	251	Suspected infection (Sepsis, severe sepsis, or septic shock) in ICU patients	American College of Chest Physicians / Society of Critical Care Medicine (ACCP/SCCM) Consensus Conference definitions	Initiation guidance: PCT <0.25 μg/L discouraged; >1 μg/L recommended	Antibiotic consumption (treatment days, DDD/100 ICU days)
Najafi 2015	Randomized controlled trial (RCT)	ICU, single-center, single-blind	Iran	December 2012 to December 2013	Hospital stay	60	30	30	SIRS in ICU patients	SIRS criteria (≥2 SIRS criteria)	Initiation guidance: PCT <0.5 ng/mL → discourage; 0.5–2 ng/mL → monitor; >2 ng/mL → treat	Antibiotic use (total exposure days)
Nazer 2024 (Pro-Can Study)	Randomized, controlled, single-blinded trial	Comprehensive Cancer Hospital ICU, Jordan	Jordan	June 2019 to September 2023	Up to 28 days	156	78	78	Critically ill cancer patients with Sepsis	SEPSIS-3 criteria	Algorithm with thresholds: <0.1 ng/mL (stop if low infection likelihood), 0.1–0.25 ng/mL (reassess), ≥0.5 ng/mL (continue until <0.1 ng/mL or >90% drop)	Time to antibiotic cessation
Nobre 2008	Randomized, controlled, open trial	University hospital ICU, Switzerland	Switzerland	February 2006 to April 2007	28 days	79	39	40	Severe sepsis or septic shock	ACCP/SCCM criteria	Stop if PCT ↓ ≥90% from peak or <0.25 μg/L (if baseline ≥1 μg/L, assess at D5; if <1 μg/L, assess at D3, stop if <0.1 μg/L)	Duration of antibiotic therapy for the first infection episode
Schroeder 2009	Prospective randomized controlled trial (RCT)	Surgical ICU, single-center	Germany	October 2006 and April 2007	Hospital stay	27	14	13	Severe sepsis after abdominal surgery	ACCP/SCCM 1992 criteria	Discontinue if PCT <1 ng/ml or decreased to <35% of the initial within 3 days	Duration of antibiotic treatment
Shehabi 2014	Prospective RCT, single-blind, multicenter	Mixed ICUs (11 centers)	Australia	March 2011 and December 2012	90 days	400	200	200	Undifferentiated infection/suspected sepsis	Sepsis-2 (2001 criteria)	Stop if PCT <0.1 ng/ml, or 0.1–0.25 ng/ml if infection unlikely, or >90% drop from baseline	Antibiotic treatment days at day 28
Vishalashi 2021	Prospective RCT, non-blinded, single-center	Mixed adult ICU	India	September 2018 to February 2020	ICU stay, 28-day readmission	90	45	45	Sepsis/septic shock	Sepsis-3 criteria	Stop if PCT <0.1 ng/ml or >80% decline from baseline	Duration of antibiotic therapy (days)

### Primary Effectiveness – All-cause death

The primary efficacy endpoint of all-cause mortality was evaluated across sixteen RCTs that enrolled 7966 patients, of whom 3,985 were allocated to a PCT-guided strategy and 3,981 to standard care. The pooled effect estimate using a random-effects DerSimonian–Laird model demonstrated no statistically significant difference in all-cause mortality between groups (RR = 0.99, 95% CI 0.92 to 1.06, p = 0.72). Between-study heterogeneity was negligible, with no evidence of statistical inconsistency (τ^2^ = 0.00; I^2^ = 0.0%; Cochran's Q(15) = 7.40, p = 0.95) (Figure 3). Leave-one-out sensitivity analyses confirmed the robustness of the pooled estimate, with sequential omission of individual trials having no impact on the direction, magnitude, or statistical significance of the overall effect (Supplementary Figure 1). Additionally, subgroup analyses stratified by baseline SOFA score demonstrated no statistically significant differences in all-cause mortality across severity strata, including patients with SOFA scores 0–6 (RR = 0.90, 95% CI 0.79 to 1.03; p = 0.11), SOFA scores 7–9 (RR = 1.06, 95% CI 0.93 to 1.22; p = 0.36), and SOFA ≥10 (RR = 0.97, 95% CI 0.83 to 1.14; p = 0.74). The test for subgroup differences was not statistically significant (Qb(2) = 3.07; p = 0.22), indicating no evidence of effect modification by baseline SOFA score (Supplementary Figure 2). Subgroup analyses according to mortality time-point demonstrated consistent, non-significant effects across all predefined categories, including ICU mortality (RR = 1.08, 95% CI 0.83 to 1.41; p = 0.56), in-hospital mortality (RR = 1.07, 95% CI 0.81 to 1.41; p = 0.63), 28-day mortality (RR = 0.95, 95% CI 0.87 to 1.04; p = 0.31), 60-day mortality (RR = 1.05, 95% CI 0.92 to 1.20; p = 0.49), and 90-day mortality (RR = 1.00, 95% CI 0.89 to 1.13; p = 0.96). There was no evidence of subgroup heterogeneity (Qb(4) = 2.07; p = 0.72) (Supplementary Figure 3). Moreover, TSA demonstrated that the cumulative Z-curve did not cross monitoring boundaries for benefit or harm and remained within the futility region. Despite an accrued sample size close to the RIS, the evidence remains insufficient to draw firm conclusions regarding a small-to-moderate mortality effect (Figure 4). The L'Abbé plot demonstrated that study estimates clustered around the line of no effect, indicating comparable event risks between the PCT-guided and control groups across included trials (Supplementary Figure 4). Visual inspection of the funnel plot showed no major asymmetry, with studies distributed reasonably around the pooled effect estimate, suggesting no strong evidence of publication bias (Supplementary Figure 5).

**Fig. 3.**
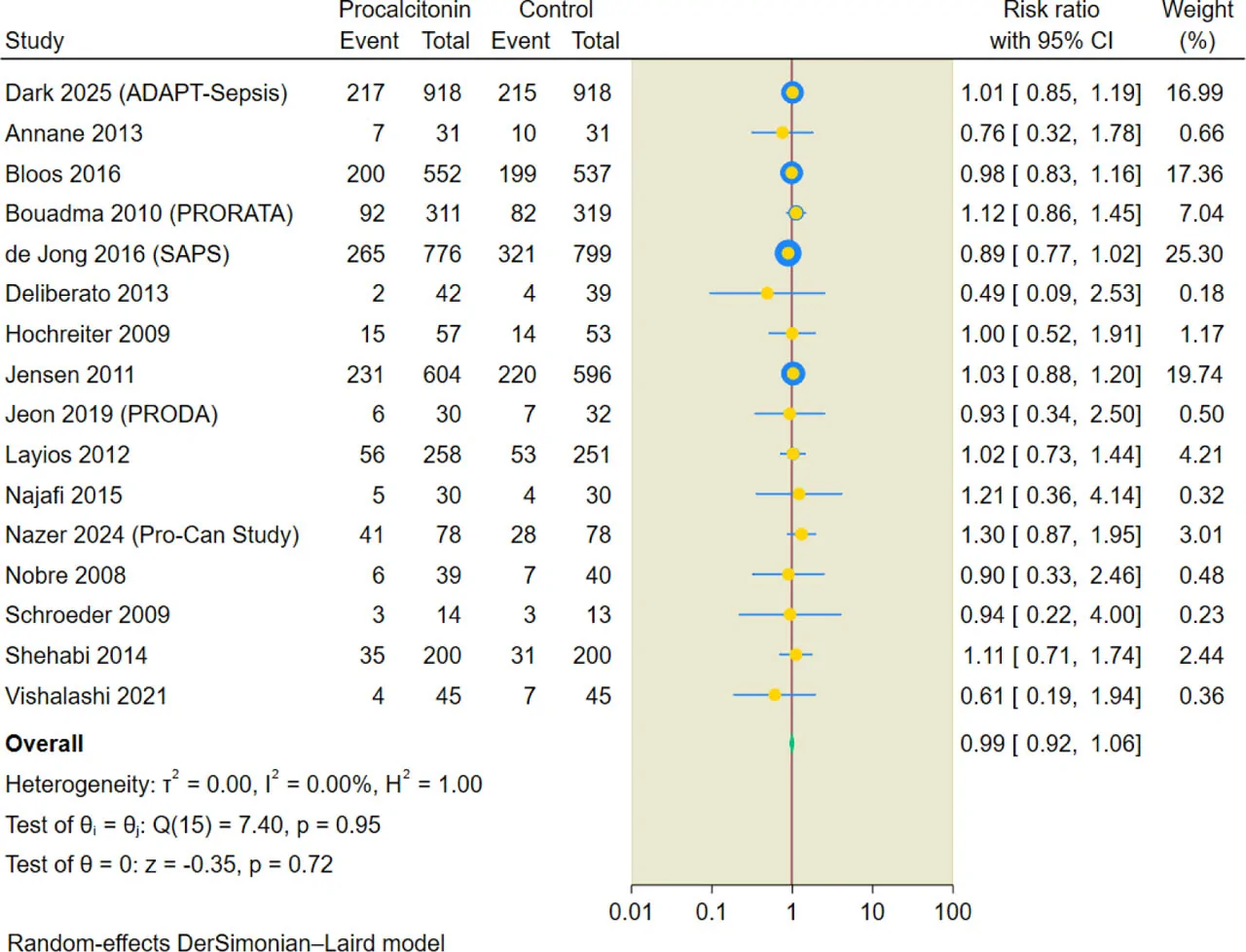
Forest plot of all-cause mortality

**Fig. 4.**
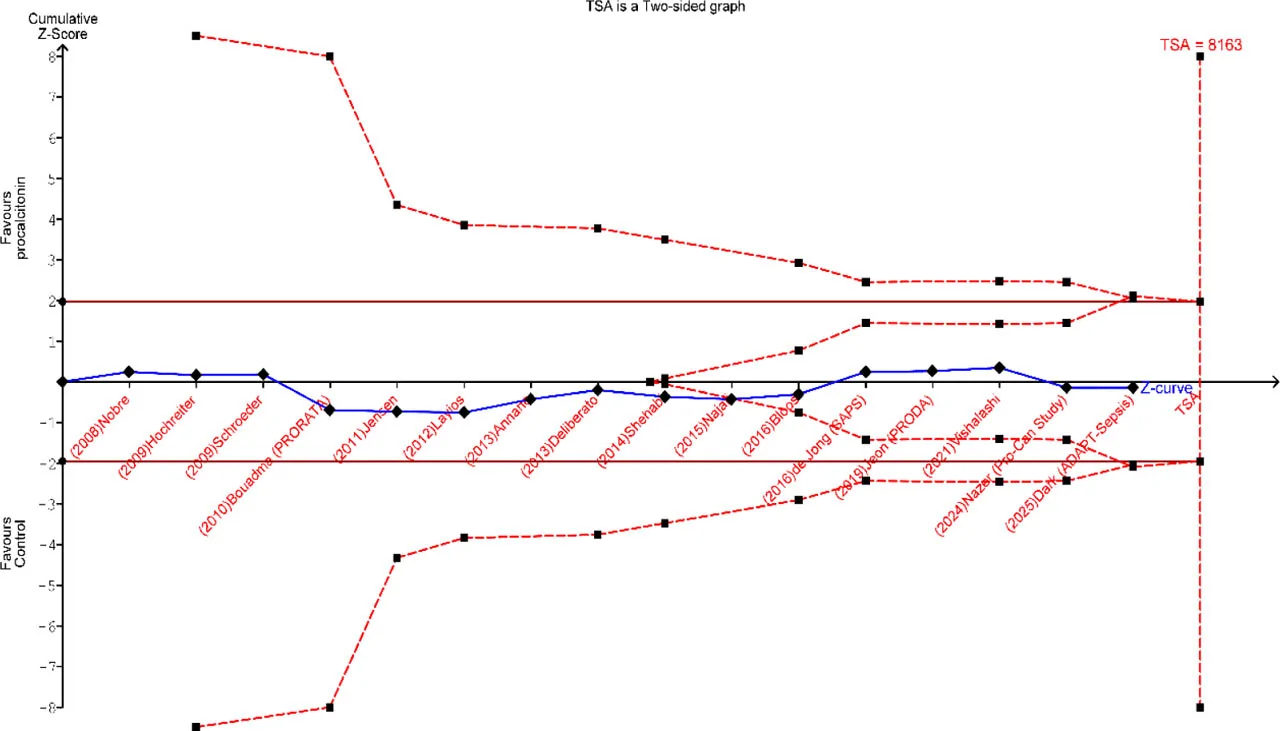
Trial sequential analysis of all-cause mortality

Meta-regression analyses showed no significant association between the treatment effect on all-cause mortality and any of the evaluated covariates, including age, sex distribution, and baseline illness severity (SOFA score and APACHE II score). Similarly, no significant effect modification was observed according to the presumed site of infection, including respiratory tract, intra-abdominal, urinary tract, skin and soft tissue, and catheter-related infections. Overall, these findings suggest that the effect of PCT-guided therapy on all-cause mortality was consistent across the examined study-level characteristics (Supplementary Table 2).

### Primary Effectiveness – Duration of Antibiotic Therapy

The effectiveness outcome of duration of antibiotic therapy was evaluated across fourteen RCTs reporting antibiotic exposure in patients managed with a PCT–guided strategy compared with standard care. Using a random-effects DerSimonian–Laird model, PCT-guided therapy was associated with a statistically significant reduction in antibiotic duration (MD = −1.51 days, 95% CI −2.24 to −0.78; p < 0.0001). Substantial between-study heterogeneity was observed (τ^2^ = 1.39; I^2^ = 87.6%; Cochran's Q(13) = 104.72, p < 0.001) (Figure 5). Leave-one-out sensitivity analyses confirmed the robustness of the pooled estimate, with sequential omission of individual trials having no material impact on the direction or statistical significance of the overall effect (Supplementary Figure 6). Furthermore, subgroup analyses stratified by baseline SOFA score demonstrated a significant reduction in antibiotic duration among patients with lower to intermediate disease severity, including those with SOFA scores 0–6 (MD = −2.51 days, 95% CI −3.76 to −1.26; p < 0.0001) and SOFA scores 7–9 (MD = −1.64 days, 95% CI −2.51 to −0.76; p < 0.0001). In contrast, no statistically significant reduction was observed among patients with SOFA ≥10 (MD = −0.44 days, 95% CI −1.50 to 0.62; p = 0.42). The test for subgroup differences was statistically significant (Qb(2) = 6.43; p = 0.04) (Supplementary Figure 7). By TSA, the cumulative Z-curve crossed both the conventional boundary for statistical significance and the trial sequential monitoring boundary for benefit, supporting a statistically reliable reduction in antibiotic duration with PCT-guided therapy. The accrued sample size approached the RIS, indicating that the available evidence is sufficient and conclusive to support this finding (Figure 6). Heterogeneity was further explored using the Galbraith plot, which identified two studies lying outside the 95% confidence limits of the regression line, indicating that these trials contributed to between-study heterogeneity (Supplementary Figure 8). Potential publication bias was assessed using a funnel plot, which demonstrated marked asymmetry, with three studies imputed by the trim-and-fill method to restore symmetry (Supplementary Figure 9A-B). The adjusted analysis did not materially alter the overall effect estimate, suggesting that the observed asymmetry may be attributable to limited available literature and underlying clinical heterogeneity rather than true publication bias.

**Fig. 5.**
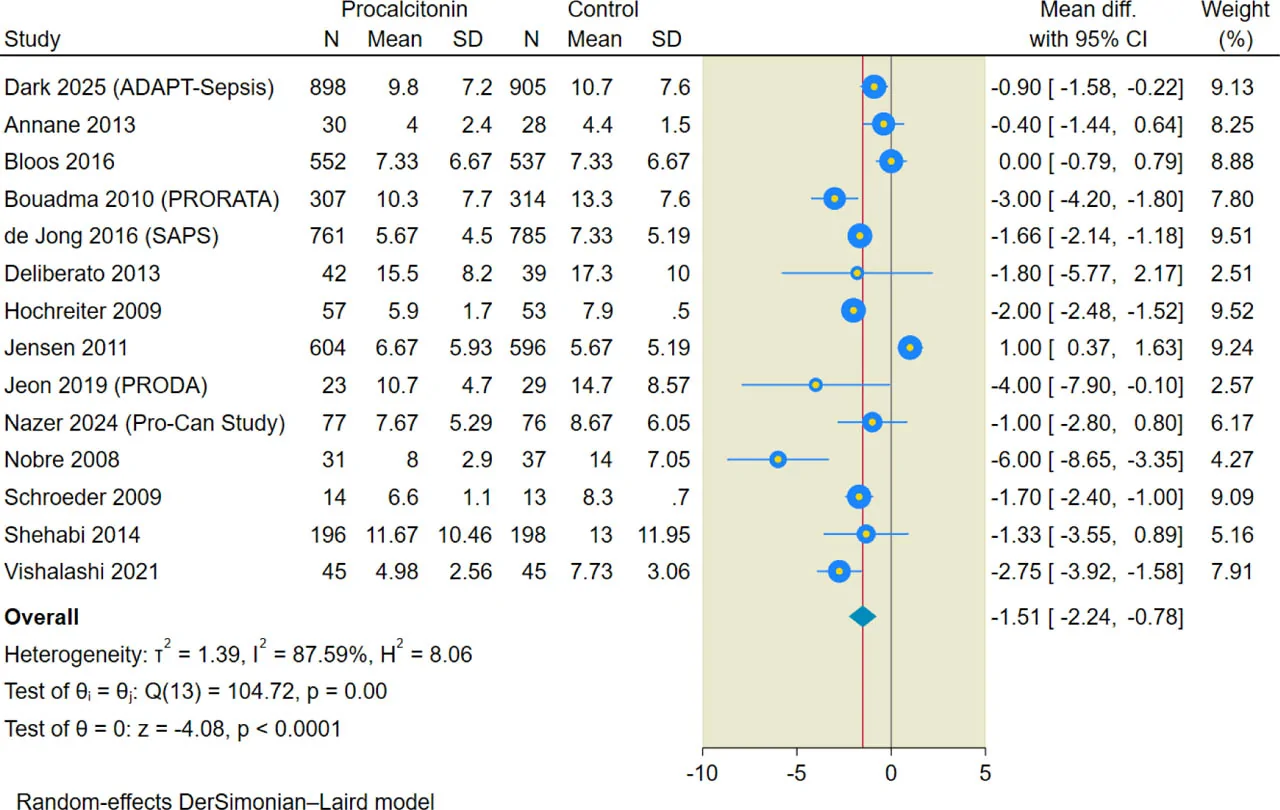
Forest plot of duration of antibiotic therapy

**Fig. 6.**
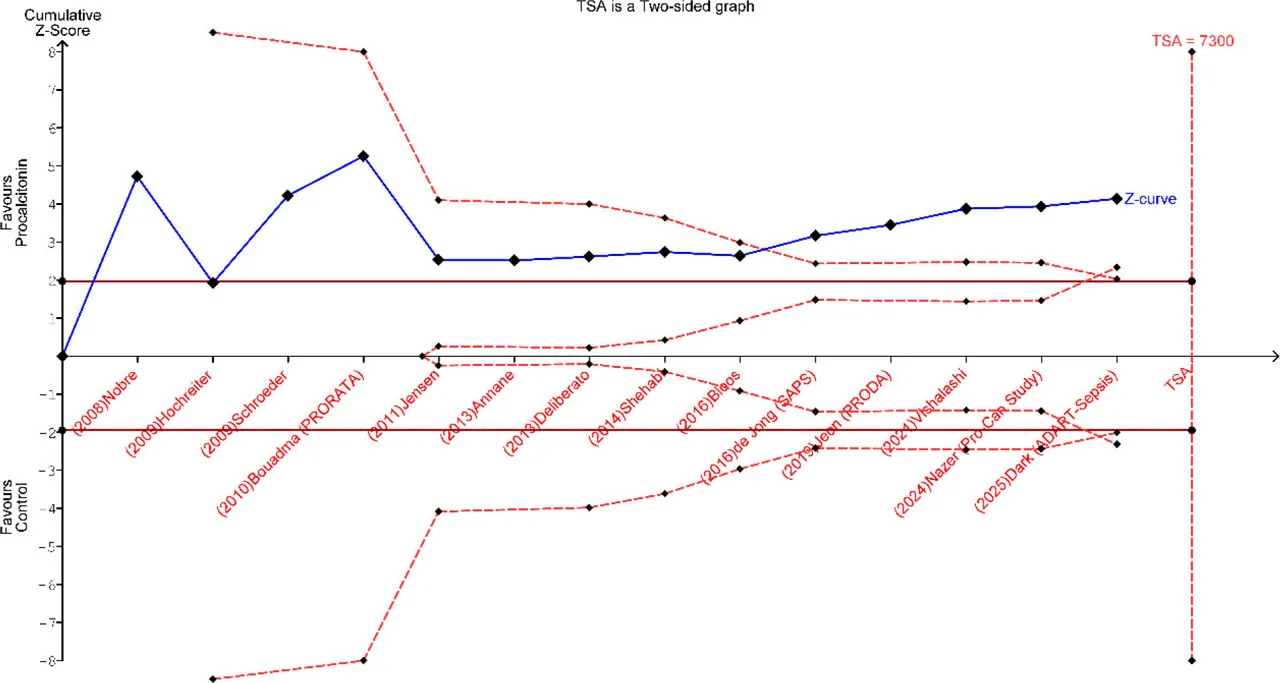
Trial sequential analysis of duration of antibiotic therapy

Meta-regression analyses identified several study-level covariates associated with the effect on the duration of antibiotic therapy. Sex distribution (female proportion) and baseline illness severity, as measured by the SOFA score, were significant moderators of the treatment effect, indicating that studies with a higher proportion of female patients and greater illness severity tended to report longer durations of antibiotic therapy. In contrast, age, male sex distribution, and APACHE II score were not significantly associated with the treatment effect. Likewise, no significant effect modification was observed according to the presumed site of infection, including respiratory tract, intra-abdominal, urinary tract, skin and soft tissue, and catheter-related infections (Supplementary Table 3).

### Secondary Clinical Outcomes

Using a random-effects DerSimonian–Laird model, pooled analyses demonstrated no statistically significant differences between PCT-guided strategies and standard care across secondary clinical and safety outcomes. Specifically, PCT-guided therapy was associated with a non-significant trend toward higher infection relapse (RR = 1.35, 95% CI 0.98 to 1.85; p = 0.06), with negligible between-study heterogeneity (I^2^ = 0.0%; τ^2^ = 0.00; p = 0.70) (Supplementary Figure 10). Similarly, no difference was observed in the occurrence of new infection or superinfection (RR = 1.00, 95% CI 0.82 to 1.22; p = 0.98), with moderate heterogeneity noted across studies (I^2^ = 33.2%; τ^2^ = 0.01; p = 0.20) (Supplementary Figure 11). In addition, clinical cure rates were comparable between groups (RR = 1.02, 95% CI 0.81 to 1.28; p = 0.90), with no evidence of heterogeneity (I^2^ = 0.0%; τ^2^ = 0.00; p = 0.51) (Supplementary Figure 12).

### Secondary Efficacy Outcomes

Antimicrobial-free days were reported across seven trials involving 1954 patients receiving a PCT-guided strategy and 1975 patients receiving standard care. The pooled estimate based on the random-effects DerSimonian–Laird method demonstrated that PCT-guided therapy was associated with a significant increase in antimicrobial-free days compared with standard care (MD = 1.19 days, 95% CI 0.40 to 1.98; p < 0.0001), although substantial heterogeneity was observed across studies (I^2^ = 69.2%) (Supplementary Figure 13). In contrast, no statistically significant differences were observed in length of hospital stay (MD = −0.41 days, 95% CI −2.15 to 1.33; p = 0.64; I^2^ = 65.0%) or length of ICU stay (MD = −0.25 days, 95% CI −0.97 to 0.48; p = 0.51; I^2^ = 72.6%) (Supplementary Figure 14). The GRADE assessment (Supplementary Table 4) indicated high-certainty evidence for all-cause mortality and new infection or superinfection; moderate-certainty evidence for duration of antibiotic therapy, infection relapse, clinical cure, and antimicrobial-free days; and low-certainty evidence for hospital or ICU length of stay.

## Discussion

In this systematic review and meta-analysis of RCTs conducted in critically ill adult patients admitted to the ICU, we comprehensively evaluated the effectiveness and safety of PCT-guided antibiotic strategies compared with standard care. Overall, PCT-guided therapy was not associated with a statistically significant reduction in all-cause mortality at any evaluated follow-up time point. However, PCT-guided strategies were associated with a shorter duration of antibiotic therapy and more antibiotic-free days, without clear evidence of increased infection relapse, superinfection, or reduced clinical cure rates. These findings support the interpretation that PCT-guided therapy functions primarily as an antimicrobial stewardship intervention rather than a survival-improving treatment.

Our analysis demonstrated no significant difference in all-cause mortality between PCT-guided therapy and standard care, despite the biological rationale supporting PCT as a marker of bacterial infection severity and resolution. This finding was consistent across all predefined mortality time points, including ICU, in-hospital, 28-day, 60-day, and 90-day mortality, and across illness severity strata defined by SOFA score. The absence of heterogeneity in these analyses strengthens the robustness of this conclusion. According to GRADE assessment, this high-certainty evidence increases confidence that PCT-guided therapy does not influence survival in critically ill patients. Mortality in critically ill patients with sepsis or suspected infection is multifactorial and influenced by host factors, comorbidities, severity of organ dysfunction, adequacy of initial resuscitation, timeliness of source control, and overall quality of supportive care, rather than antibiotic duration alone [1,37]. PCT-guided strategies inform decisions regarding antibiotic discontinuation rather than early initiation of empirical therapy, which are more likely to influence survival in septic patients. These conclusions are further strengthened by TSA, which demonstrated that the cumulative evidence did not cross boundaries for benefit or harm and entered the futility region, suggesting that further trials are unlikely to reveal a clinically meaningful mortality effect of PCT-guided therapy in the ICU population. Notably, these findings are strongly reinforced by the recently published ADAPT-Sepsis, which includes the largest ICU-specific biomarker-guided antibiotic trial to date. ADAPT-Sepsis demonstrated that daily PCT-guided antibiotic discontinuation was noninferior to standard care for both 28-day and 90-day all-cause mortality, despite enrolling a population with substantial illness severity. The convergence of results between ADAPT-Sepsis and the present meta-analysis provides compelling contemporary evidence that PCT-guided strategies do not confer a survival advantage in critically ill patients [15].

In contrast to mortality outcomes, PCT-guided therapy was associated with a significant reduction in the duration of antibiotic therapy, with a mean decrease of approximately 1.5 days compared with standard care [1]. The TSA also confirmed this finding by statistically supporting a sufficient accumulation of information. Although substantial heterogeneity was observed, sensitivity analyses demonstrated that the magnitude of effect was robust to the exclusion of individual studies. From a clinical perspective, modest reductions in antibiotic duration may translate into substantial benefits at both patient and system levels, including reduced risk of antibiotic-associated adverse events, decreased selective pressure for antimicrobial resistance, and lower healthcare costs [38]. These benefits are particularly relevant in ICU settings, where the use of broad-spectrum antibiotics is common, and antimicrobial resistance poses a growing threat. These benefits are particularly relevant in ICU settings, where the use of broad-spectrum antibiotics is common, and antimicrobial resistance poses a growing threat. The stewardship benefit observed in this meta-analysis is further substantiated by ADAPT-Sepsis, which reported a statistically significant reduction in total antibiotic duration of nearly one day in the PCT-guided group compared with standard care [15]. Subgroup analyses revealed that the reduction in antibiotic duration was observed among patients with low to intermediate illness severity (SOFA scores 0–6 and 7–9), whereas no significant reduction was observed in patients with SOFA scores ≥10. This finding suggests that PCT-guided strategies may be less effective in patients with severe organ dysfunction, in whom persistent inflammation, secondary infections, or clinician concern regarding clinical instability may override biomarker-based recommendations.

In this analysis, PCT-guided therapy was noninferior to the standard of care for new infections, superinfections, or failure to achieve clinical cure. Although a non-significant trend toward higher infection relapse was observed, the absolute risk difference was small, heterogeneity was negligible, and confidence intervals included unity, suggesting that this finding should be interpreted cautiously. Furthermore, PCT-guided therapy was associated with a significant increase in antibiotic-free days, reinforcing its role in reducing unnecessary antimicrobial exposure without compromising patient safety. No significant differences were observed in ICU or hospital length of stay, indicating that reduced antibiotic use did not adversely affect short-term clinical recovery or resource utilization.

Substantial heterogeneity was observed for antibiotic duration and some secondary outcomes. This variability likely reflects differences in PCT algorithms, cutoff thresholds, frequency of measurement, adherence to protocols, baseline illness severity, and local antimicrobial practices across trials. Galbraith plot analysis identified a small number of outlying studies that contributed disproportionately to heterogeneity; however, excluding these studies did not materially alter the pooled estimates. Potential publication bias was suggested by funnel plot asymmetry for antibiotic duration; however, trim-and-fill analysis did not change the overall effect size. This asymmetry may reflect clinical heterogeneity or selective reporting of stewardship-related outcomes rather than true publication bias.

The findings of the present meta-analysis are broadly consistent with the existing body of evidence evaluating PCT-guided antibiotic strategies in critically ill patients. Several large RCTs conducted in ICU populations have consistently demonstrated that PCT-guided algorithms reduce antibiotic exposure without a corresponding increase in mortality. Notably, the PRORATA trial, which enrolled ICU patients with suspected bacterial infections, showed a significant reduction in antibiotic duration in the PCT-guided group, while mortality at 28 and 60 days remained comparable to standard care [26]. Similarly, the SAPS trial, one of the largest ICU-based RCTs to date, confirmed that PCT-guided antibiotic discontinuation safely reduced antibiotic use without increasing 28-day or 1-year mortality, despite enrolling a population with substantial illness severity [5]. These findings have been corroborated by multiple meta-analyses. Earlier pooled analyses, including those by Schuetz et al. and Papp et al., reported significant reductions in antibiotic duration and exposure among critically ill patients managed with PCT guidance, with no adverse impact on survival [39,40]. However, many of these analyses combined heterogeneous patient populations, including non-ICU settings, or incorporated non-randomized studies, limiting the strength of causal inference. In contrast, our analysis exclusively included RCTs conducted in ICU settings, thereby minimizing clinical and methodological heterogeneity related to care environments. The inclusion of ADAPT-Sepsis substantially strengthens the evidence base by providing high-certainty RCT data that align closely with our pooled estimates and reinforces the interpretation of PCT guidance as a stewardship rather than a disease-modifying intervention [15].

More recent meta-analyses have suggested a potential mortality benefit of PCT-guided therapy in critically ill patients; however, these findings remain inconsistent and controversial [41,42,43]. Differences in included study designs, outcome definitions, and follow-up durations likely contribute to these discrepancies. Our subgroup analyses further refine the interpretation of prior evidence by demonstrating that reductions in antibiotic duration are primarily driven by patients with low to intermediate illness severity, as defined by baseline SOFA scores. This observation aligns with post hoc analyses of major RCTs, which have suggested that clinicians are more willing to adhere to PCT-guided discontinuation algorithms in clinically stable patients [44]. Such behavior may partially explain why antibiotic duration was not significantly reduced among patients with SOFA scores ≥10 in our analysis and underscores the importance of clinical context when applying biomarker-guided strategies. These findings reinforce that PCT-guided antibiotic therapy is best viewed as a stewardship intervention rather than a disease-modifying treatment [45].

### Clinical implications

Taken together, these findings support the use of PCT-guided antibiotic strategies as a safe, evidence-based antimicrobial stewardship intervention in critically ill patients, particularly among those with low to moderate illness severity. In this population, PCT guidance facilitates earlier discontinuation of antibiotics without compromising clinical outcomes, thereby reducing unnecessary antimicrobial exposure and its associated risks. However, the absence of a mortality benefit indicates that PCT-guided therapy should not be expected to improve survival in the ICU setting, where outcomes are predominantly driven by disease severity, organ dysfunction, source control, and overall quality of supportive care. The GRADE evidence profile supports strong confidence in this recommendation for stewardship purposes, while discouraging expectations of survival benefit. Importantly, PCT should not be applied as a standalone decision-making tool or used to override sound clinical judgment, especially in patients with severe organ failure, persistent hemodynamic instability, or unresolved infectious sources [46]. Rather, the greatest clinical value of PCT appears to lie in its integration into structured antimicrobial stewardship protocols, where biomarker trends are interpreted in line with clinical trajectory, microbiological data, and complementary inflammatory markers, enabling individualized and context-sensitive antibiotic management [47].

### Strengths and limitations

This systematic review and meta-analysis have several important strengths. First, the analysis was restricted exclusively to RCTs conducted in adult ICU populations, thereby minimizing confounding related to study design and care setting and strengthening causal inference. Second, the large, pooled sample size and inclusion of nearly 8,000 critically ill patients provide substantial statistical power to detect clinically meaningful effects, particularly for stewardship-related outcomes. Third, all analyses were performed according to the intention-to-treat principle and adhered to rigorous methodological standards. Fourth, the application of TSA represents a major methodological strength, as it allowed evaluation of the conclusiveness of the evidence and mitigation of risks associated with random error and repetitive significance testing. Finally, prespecified subgroup analyses based on illness severity and mortality time points enhance the clinical interpretability of the findings and provide nuanced insights into patient populations most likely to benefit from PCT-guided strategies.

Several limitations merit consideration. Despite the exclusive inclusion of RCTs, substantial clinical heterogeneity was observed for certain outcomes, particularly duration of antibiotic therapy and length of stay. This heterogeneity likely reflects variability in PCT algorithms, cutoff thresholds, frequency of biomarker measurement, adherence to protocol recommendations, baseline illness severity, and institutional antimicrobial stewardship practices across studies. Second, although funnel plot asymmetry was observed for antibiotic duration, adjusted analyses using trim-and-fill methods did not materially alter the pooled effect estimates, suggesting that the observed asymmetry may be attributable to limited study numbers or underlying clinical heterogeneity rather than true publication bias. Nevertheless, the possibility of small study effects cannot be fully excluded. Third, the absence of individual patient-level data precluded more granular exploration of effect modification by factors such as infection source, pathogen type, appropriateness of initial empirical therapy, or dynamic changes in organ dysfunction. Fourth, clinician adherence to PCT-guided algorithms varied across trials and was incompletely reported, potentially attenuating the observed effects and limiting generalizability to real-world settings. Finally, while TSA strengthened inference regarding mortality and antibiotic duration, it does not eliminate uncertainty related to long-term outcomes or rare adverse events, which remain insufficiently reported in the existing literature.

### Future research

Future studies should focus on individual patient data meta-analyses to better identify subgroups most likely to benefit from PCT-guided strategies, standardization of PCT thresholds and protocols, and evaluation of combined biomarker approaches. Trials targeting specific ICU populations, such as immunocompromised patients or those with septic shock, may further refine the role of PCT-guided antibiotic management.

## Conclusion

Overall, PCT-guided therapy was not associated with a statistically significant reduction in all-cause mortality at any evaluated follow-up time point, indicating no survival benefit in the available randomized evidence. However, PCT-guided strategies were associated with a shorter duration of antibiotic therapy and more antibiotic-free days, without clear evidence of increased infection relapse, superinfection, or reduced clinical cure rates. These findings support the interpretation that PCT-guided therapy functions primarily as an antimicrobial stewardship intervention rather than a survival-improving treatment.

## Supplementary Material

Supplementary Material Details

## References

[1] Shehabi Y, Sterba M, Garrett PM (2014). Procalcitonin algorithm in critically ill adults with undifferentiated infection or suspected sepsis. A randomized controlled trial. Am J Respir Crit Care Med..

[2] Rhodes A, Evans LE, Alhazzani W (2017). Surviving Sepsis Campaign: International Guidelines for Management of Sepsis and Septic Shock: 2016. Intensive Care Med.

[3] Evans L, Rhodes A, Alhazzani W (2021). Surviving sepsis campaign: international guidelines for management of sepsis and septic shock 2021. Intensive Care Med.

[4] Van Boeckel TP, Gandra S, Ashok A (2014). Global antibiotic consumption 2000 to 2010: an analysis of national pharmaceutical sales data. Lancet Infect Dis.

[5] de Jong E, van Oers JA, Beishuizen A, Vos P, Vermeijden WJ, Haas LE (2016). Efficacy and safety of procalcitonin guidance in reducing the duration of antibiotic treatment in critically ill patients: a randomised, controlled, open-label trial. Lancet Infect Dis..

[6] Schuetz P, Christ-Crain M, Thomann R (2009). Effect of procalcitonin-based guidelines vs standard guidelines on antibiotic use in lower respiratory tract infections: the ProHOSP randomized controlled trial. JAMA.

[7] Prestinaci F, Pezzotti P, Pantosti A (2015). Antimicrobial resistance: a global multifaceted phenomenon. Pathog Glob Health.

[8] Huttner A, Harbarth S, Carlet J (2013). Antimicrobial resistance: a global view from the 2013 World Healthcare-Associated Infections Forum. Antimicrob Resist Infect Control..

[9] Vincent JL, Teixeira L (2014). Sepsis biomarkers. Value and limitations. Am J Respir Crit Care Med..

[10] Assicot M, Gendrel D, Carsin H, Raymond J, Guilbaud J, Bohuon C (1993). High serum procalcitonin concentrations in patients with sepsis and infection. Lancet.

[11] Nijsten MW, Olinga P, The TH (2000). Procalcitonin behaves as a fast responding acute phase protein in vivo and in vitro. Crit Care Med.

[12] Harbarth S, Holeckova K, Froidevaux C (2001). Diagnostic value of procalcitonin, interleukin-6, and interleukin-8 in critically ill patients admitted with suspected sepsis. Am J Respir Crit Care Med.

[13] Simon L, Gauvin F, Amre DK, Saint-Louis P, Lacroix J (2004). Serum procalcitonin and C-reactive protein levels as markers of bacterial infection: a systematic review and meta-analysis. Clin Infect Dis.

[14] Oczkowski S, Alshamsi F, Belley-Cote E (2022). Surviving Sepsis Campaign Guidelines 2021: highlights for the practicing clinician. Pol Arch Intern Med.

[15] Dark P, Hossain A, McAuley DF (2025). Biomarker-Guided Antibiotic Duration for Hospitalized Patients With Suspected Sepsis: The ADAPT-Sepsis Randomized Clinical Trial. JAMA.

[16] Page MJ, McKenzie JE, Bossuyt PM (2021). The PRISMA 2020 statement: an updated guideline for reporting systematic reviews. BMJ..

[17] Cumpston M, Li T, Page MJ (2019). Updated guidance for trusted systematic reviews: a new edition of the Cochrane Handbook for Systematic Reviews of Interventions. Cochrane Database Syst Rev.

[18] Ouzzani M, Hammady H, Fedorowicz Z, Elmagarmid A (2016). Rayyan-a web and mobile app for systematic reviews. Syst Rev.

[19] Sterne JAC, Savović J, Page MJ (2019). RoB 2: a revised tool for assessing risk of bias in randomised trials. BMJ..

[20] Kang H (2021). Trial sequential analysis: novel approach for meta-analysis. Anesth Pain Med (Seoul).

[21] Wetterslev J, Jakobsen JC, Gluud C (2017). Trial Sequential Analysis in systematic reviews with meta-analysis. BMC Med Res Methodol.

[22] Riberholt CG, Olsen MH, Gluud C (2024). Research Note: Trial sequential analysis in systematic reviews with meta-analysis. J Physiother.

[23] Alonso-Coello P, Schünemann HJ, Moberg J (2016). GRADE Evidence to Decision (EtD) frameworks: a systematic and transparent approach to making well informed healthcare choices. 1: Introduction. BMJ..

[24] Annane D, Maxime V, Faller JP (2013). Procalcitonin levels to guide antibiotic therapy in adults with non-microbiologically proven apparent severe sepsis: a randomised controlled trial. BMJ Open.

[25] Bloos F, Trips E, Nierhaus A (2016). Effect of Sodium Selenite Administration and Procalcitonin-Guided Therapy on Mortality in Patients With Severe Sepsis or Septic Shock: A Randomized Clinical Trial. JAMA Intern Med.

[26] Bouadma L, Luyt CE, Tubach F (2010). Use of procalcitonin to reduce patients' exposure to antibiotics in intensive care units (PRORATA trial): a multicentre randomised controlled trial. Lancet.

[27] Deliberato RO, Marra AR, Sanches PR (2013). Clinical and economic impact of procalcitonin to shorten antimicrobial therapy in septic patients with proven bacterial infection in an intensive care setting. Diagn Microbiol Infect Dis.

[28] Hochreiter M, Köhler T, Schweiger AM (2009). Procalcitonin to guide duration of antibiotic therapy in intensive care patients: a randomized prospective controlled trial. Crit Care.

[29] Jensen JU, Hein L, Lundgren B (2011). Procalcitonin-guided interventions against infections to increase early appropriate antibiotics and improve survival in the intensive care unit: a randomized trial. Crit Care Med.

[30] Jeon K, Suh JK, Jang EJ (2019). Procalcitonin-Guided Treatment on Duration of Antibiotic Therapy and Cost in Septic Patients (PRODA): a Multi-Center Randomized Controlled Trial. J Korean Med Sci.

[31] Layios N, Lambermont B, Canivet JL (2012). Procalcitonin usefulness for the initiation of antibiotic treatment in intensive care unit patients. Crit Care Med.

[32] Najafi A, Khodadadian A, Sanatkar M (2015). The Comparison of Procalcitonin Guidance Administer Antibiotics with Empiric Antibiotic Therapy in Critically Ill Patients Admitted in Intensive Care Unit. Acta Med Iran.

[33] Nazer LH, Awad W, Thawabieh H (2024). Procalcitonin-Guided Management and Duration of Antibiotic Therapy in Critically Ill Cancer Patients With Sepsis (Pro-Can Study): A Randomized Controlled Trial. Crit Care Explor.

[34] Nobre V, Harbarth S, Graf JD, Rohner P, Pugin J (2008). Use of procalcitonin to shorten antibiotic treatment duration in septic patients: a randomized trial. Am J Respir Crit Care Med.

[35] Schroeder S, Hochreiter M, Koehler T (2009). Procalcitonin (PCT)-guided algorithm reduces length of antibiotic treatment in surgical intensive care patients with severe sepsis: results of a prospective randomized study. Langenbecks Arch Surg.

[36] Vishalashi SG, Gupta P, Verma PK (2021). Serum Procalcitonin as a Biomarker to Determine the Duration of Antibiotic Therapy in Adult Patients with Sepsis and Septic Shock in Intensive Care Units: A Prospective Study. Indian J Crit Care Med.

[37] Ibarz M, Haas LEM, Ceccato A, Artigas A (2024). The critically ill older patient with sepsis: a narrative review. Ann Intensive Care.

[38] Mo Y, Tan WC, Cooper BS (2025). Antibiotic duration for common bacterial infections-a systematic review. JAC Antimicrob Resist.

[39] Schuetz P, Müller B, Christ-Crain M (2012). Procalcitonin to initiate or discontinue antibiotics in acute respiratory tract infections. Cochrane Database Syst Rev.

[40] Papp M, Kiss N, Baka M (2023). Procalcitonin-guided antibiotic therapy may shorten length of treatment and may improve survival-a systematic review and meta-analysis. Crit Care.

[41] Schuetz P, Wirz Y, Sager R (2018). Effect of procalcitonin-guided antibiotic treatment on mortality in acute respiratory infections: a patient level meta-analysis. Lancet Infect Dis.

[42] Lam SW, Bauer SR, Fowler R, Duggal A (2018). Systematic Review and Meta-Analysis of Procalcitonin-Guidance Versus Usual Care for Antimicrobial Management in Critically Ill Patients: Focus on Subgroups Based on Antibiotic Initiation, Cessation, or Mixed Strategies. Crit Care Med.

[43] Zhang T, Wang Y, Yang Q, Dong Y (2017). Procalcitonin-guided antibiotic therapy in critically ill adults: a meta-analysis. BMC Infect Dis.

[44] Hohn A, Balfer N, Heising B (2018). Adherence to a procalcitonin-guided antibiotic treatment protocol in patients with severe sepsis and septic shock. Ann Intensive Care.

[45] Kiya GT, Asefa ET, Abebe G, Mekonnen Z (2024). Procalcitonin Guided Antibiotic Stewardship. Biomark Insights..

[46] Gupta S, Klompas M, Rhee C (2026). Reassessing Procalcitonin-Guided Antibiotic Therapy in Critically Ill Patients With Sepsis: Lessons From the ADAPT-Sepsis Trial. Clin Infect Dis.

[47] Schuetz P (2022). How to best use procalcitonin to diagnose infections and manage antibiotic treatment. Clin Chem Lab Med.

