# Supplementary material for: Procalcitonin-guided antibiotic therapy in ICU patients with sepsis: a systematic review and meta-analysis with GRADE and trial sequential analysis
Source: J Crit Care Med (Targu Mures). 2026 Oct 30;12(4):471–86. doi: 10.62838/jccm-2026-0043 (PMC13752145; doi:10.62838/jccm-2026-0043)
Supplement: Supplementary file 1 [file jccm-2026-0043-supplementary.pdf]

DOI: 10.62838/jccm-2026-0043

Supplementary tables and figures

Supplementary Table 1: Detailed search strategy for each database

Database	Search term	Filter	Results
PubMed	(procalcitonin OR PCT) AND (sepsis OR septic OR "septic shock") AND (antibiotic* OR antimicrobial* OR "antibiotic therapy" OR "antibiotic duration" OR "antibiotic discontinuation" OR "antibiotic de-escalation")	All Filed	1,173
Scopus	(procalcitonin OR PCT) AND (sepsis OR septic OR "septic shock") AND (antibiotic* OR antimicrobial* OR "antibiotic therapy" OR "antibiotic duration" OR "antibiotic discontinuation" OR "antibiotic de-escalation")	Article title, abstract, keywords	3,552
Web of Science	(procalcitonin OR PCT) AND (sepsis OR septic OR "septic shock") AND (antibiotic* OR antimicrobial* OR "antibiotic therapy" OR "antibiotic duration" OR "antibiotic discontinuation" OR "antibiotic de-escalation")	All Filed	1,764
Cochrane library	(procalcitonin OR PCT) AND (sepsis OR septic OR "septic shock") AND (antibiotic* OR antimicrobial* OR "antibiotic therapy" OR "antibiotic duration" OR "antibiotic discontinuation" OR "antibiotic de-escalation")	All Filed	241

Supplementary Table 2: Random-Effects Meta-Regression for all-cause death

Variables		All-cause mortality	
		Coefficient	P-value
Age		-.004569	0.693
Gender	Male	.0058615	0.708
	Female	.0016247	0.738
Severity of illness	SOFA score	.0155116	0.543
	APACHE II	-.0026998	0.096
Presumed site of infection causing sepsis	Respiratory tract	-.0057397	0.704
	Intra-abdominal	.0052273	0.685
	UTI	-.0055951	0.734
	Skin and soft tissue	.0085824	0.828
	Catheter-related infection	.1453702	0.224

Supplementary Table 3: Random-Effects Meta-Regression for Duration of Antibiotic Therapy

Variables		Duration of Antibiotic Therapy	
		Coefficient	P-value
Age		.0292357	0.631
Gender	Male	.0924577	0.110
	Female	.035137	0.046
Severity of illness	SOFA score	.4041315	0.017
	APACHE II	-.0120087	0.627
Presumed site of infection causing sepsis	Respiratory tract	.0760309	0.412
	Intra-abdominal	.1406737	0.141
	UTI	-.1002509	0.167
	Skin and soft tissue	-.2803706	0.335
	Catheter-related infection	-1.582461	0.075

Supplementary Table 4: GRADE Evidence Profile

Certainty assessment							№ of patients		Effect		Certainty	Importance
№ of studies	Study design	Risk of bias				Other considerations		Control	Relative (95% CI)	Absolute (95% CI)		
All-cause mortality												
16	randomized trials	not serious	not serious	not serious	not serious	none			RR 0.99 (0.92 to 1.06)	1,000 fewer per 1,000 (from 1,000 fewer to 1,000 more)	⊕⊕⊕⊕ High	CRITICAL
Duration of antibiotic therapy												
14	randomized trials	not serious	serious[a]	not serious	not serious	none	3637	3655	-	MD 1.51 Days lower (2.24 lower to 0.78 lower)	⊕⊕⊕◯ Moderate[a]	IMPORTANT
Infection relapse												
9	randomized trials	not serious	not serious	not serious	serious[b]	none	90/2913 (3.1%)	67/2929 (2.3%)	RR 1.35 (0.98 to 1.85)	8 more per 1,000 (from 0 fewer to 19 more)	⊕⊕⊕◯ Moderate[b]	CRITICAL
New infection or superinfection												
5	randomized trials	not serious	not serious	not serious	not serious	none	459/1857 (24.7%)	460/1850 (24.9%)	RR 1.00 (0.82 to 1.22)	0 fewer per 1,000 (from 45 fewer to 55 more)	⊕⊕⊕⊕ High	IMPORTANT
Clinical cure												
4	randomized trials	not serious	not serious	not serious	serious[c]	none	85/651 (13.1%)	84/639 (13.1%)	RR 1.02 (0.81 to 1.28)	3 more per 1,000 (from 25 fewer to 37 more)	⊕⊕⊕◯ Moderate[c]	IMPORTANT
Antimicrobial-free days												
7	randomized trials	not serious	serious[d]	not serious	not serious	none	1954	1975	-	MD 1.19 days higher (0.4 higher to 1.98 higher)	⊕⊕⊕◯ Moderate[d]	IMPORTANT
Length of hospital stay												
11	randomized trials	not serious	serious[e]	not serious	serious[f]	none	2488	2509	-	MD 0.41 days lower (2.15 lower to 1.33 higher)	⊕⊕◯◯ Low[e,f]	IMPORTANT
Length of ICU stay												
16	randomized trials	not serious	serious[g]	not serious	serious[f]	none	3790	3793	-	MD 0.25 days lower (0.97 lower to 0.48 higher)	⊕⊕◯◯ Low[f,g]	IMPORTANT

CI: confidence interval; MD: mean difference; RR: risk ratio

Explanations

a. Very high heterogeneity ($I^2 \approx 87.6\%$, $\tau^2 = 1.39$; Q $p<0.0001$)
b. CI includes appreciable harm (upper CI 1.85 > 1.25) and few total events
c. CI includes potential clinically important harm (>1.25)
d. Substantial heterogeneity ($I^2 \approx 69\%$), variable magnitude across trials
e. Substantial heterogeneity ($I^2 \approx 65\%$)
f. CI includes clinically relevant reduction and increase in stay
g. High heterogeneity ($I^2 \approx 73\%$)

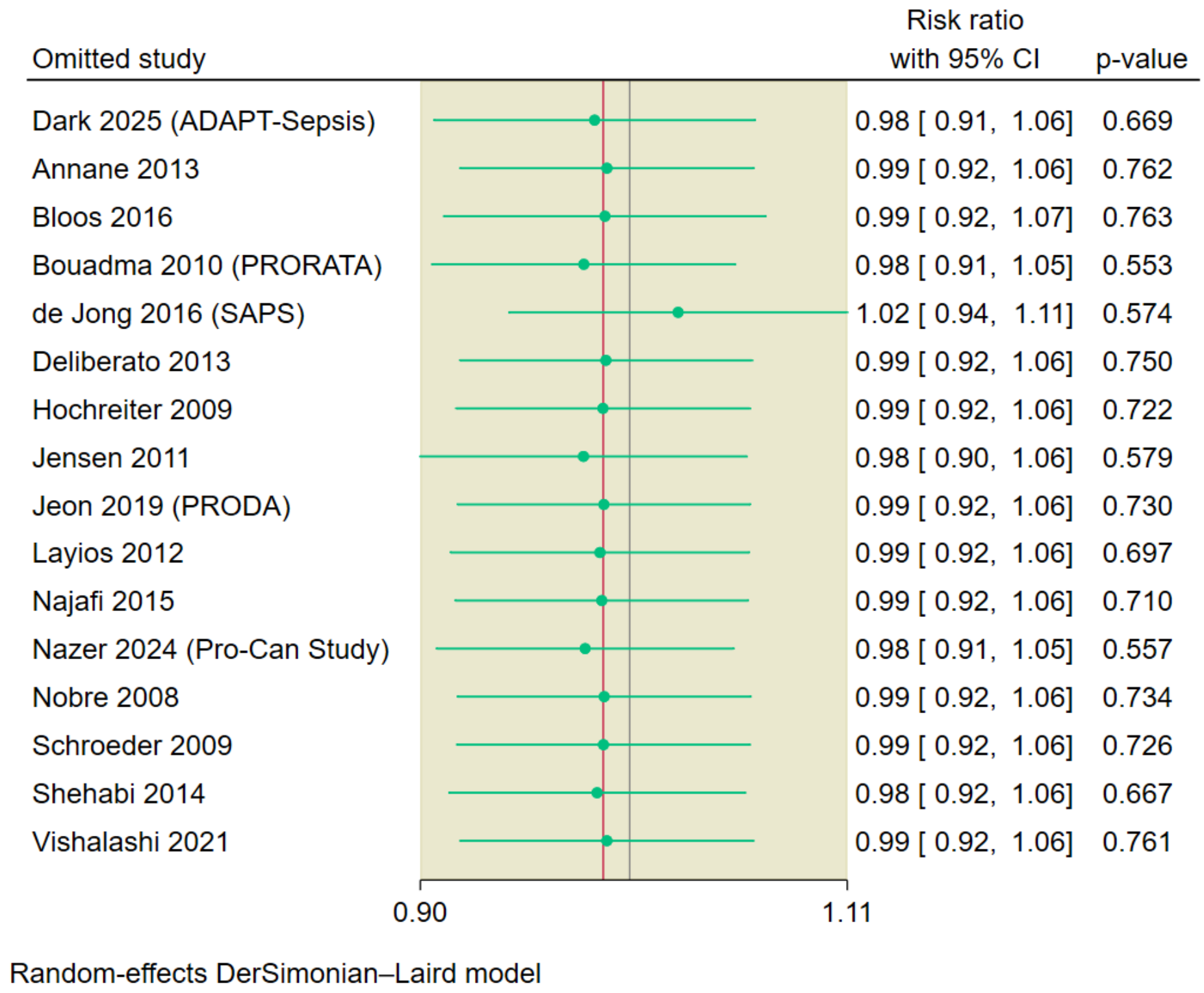

Supplementary Figure 1: Leave-one-out analysis of all-cause mortality

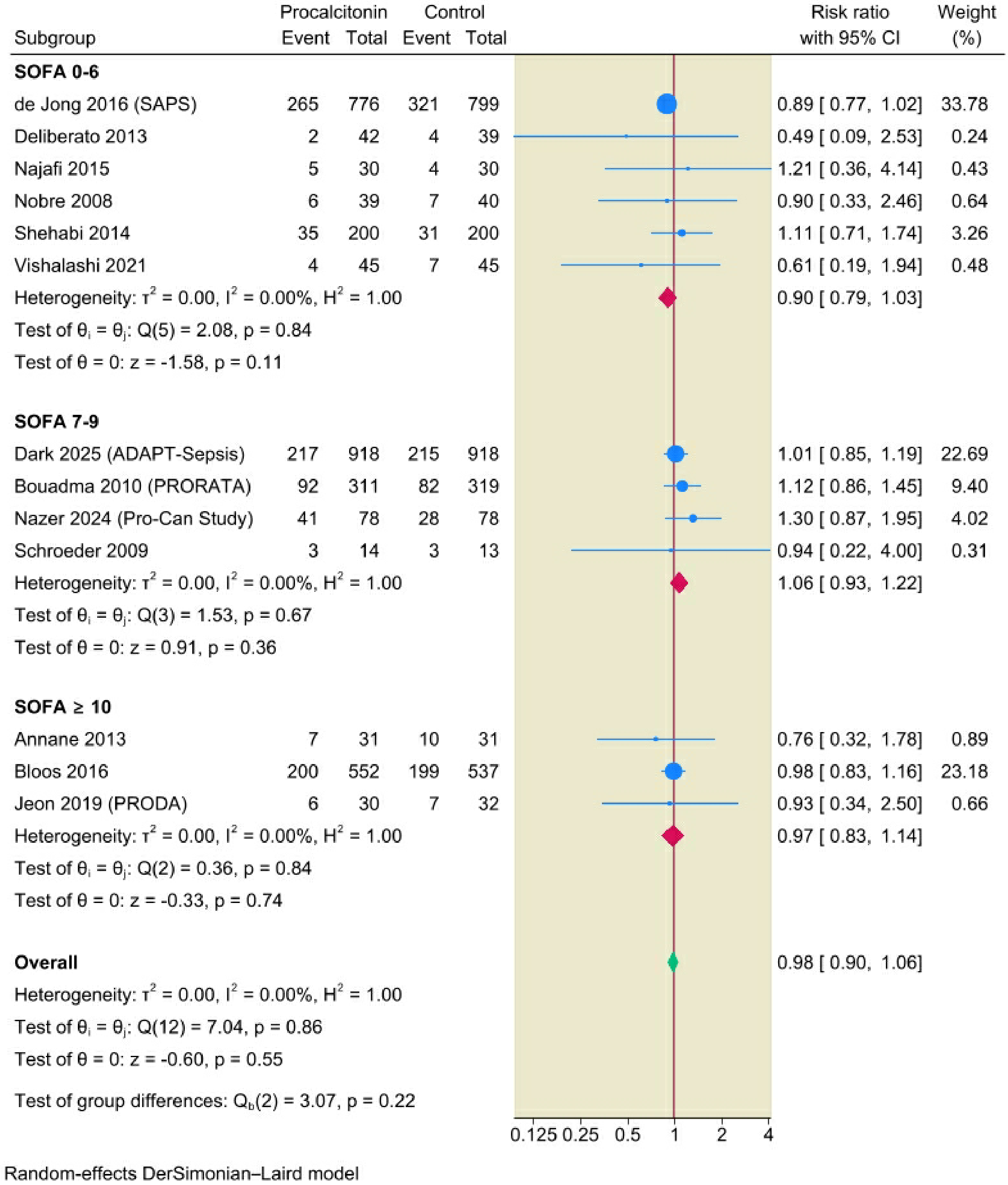

Supplementary Figure 2: Subgroup analysis of all-cause mortality stratified by baseline SOFA score.

Subgroup	Procalcitonin Event	Procalcitonin Total	Control Event	Control Total	Risk ratio with 95% CI	Weight (%)
**ICU mortality**						
Annane 2013	7	31	10	31	0.76 [0.32, 1.78]	0.48
Deliberato 2013	1	42	4	39	0.25 [0.03, 2.15]	0.08
Layios 2012	56	258	53	251	1.02 [0.73, 1.44]	3.04
Nazer 2024 (Pro-Can Study)	30	78	18	78	1.48 [0.88, 2.48]	1.33
Shehabi 2014	21	200	15	200	1.36 [0.72, 2.57]	0.88
Vishalashi 2021	4	45	7	45	0.61 [0.19, 1.94]	0.26
Heterogeneity: $\tau^2 = 0.01$, $I^2 = 8.08\%$, $H^2 = 1.09$					1.08 [0.83, 1.41]	
Test of $\theta_i = \theta_j$: Q(5) = 5.44, p = 0.36						
Test of $\theta = 0$: z = 0.58, p = 0.56						
**In-hospital mortality**						
Annane 2013	7	31	10	31	0.76 [0.32, 1.78]	0.48
Deliberato 2013	2	42	4	39	0.49 [0.09, 2.53]	0.13
Hochreiter 2009	15	57	14	53	1.00 [0.52, 1.91]	0.84
Jeon 2019 (PRODA)	6	30	7	32	0.93 [0.34, 2.50]	0.36
Najafi 2015	5	30	4	30	1.21 [0.36, 4.14]	0.23
Nazer 2024 (Pro-Can Study)	41	78	28	78	1.30 [0.87, 1.95]	2.18
Nobre 2008	6	39	7	40	0.90 [0.33, 2.46]	0.35
Schroeder 2009	3	14	3	13	0.94 [0.22, 4.00]	0.17
Heterogeneity: $\tau^2 = 0.00$, $I^2 = 0.00\%$, $H^2 = 1.00$					1.07 [0.81, 1.41]	
Test of $\theta_i = \theta_j$: Q(7) = 2.75, p = 0.91						
Test of $\theta = 0$: z = 0.48, p = 0.63						
**28-day mortality**						
Dark 2025 (ADAPT-Sepsis)	184	918	170	918	1.07 [0.88, 1.29]	9.70
Bloos 2016	140	552	149	537	0.93 [0.76, 1.14]	8.41
Bouadma 2010 (PRORATA)	65	311	64	319	1.03 [0.76, 1.42]	3.58
de Jong 2016 (SAPS)	149	776	196	799	0.82 [0.67, 0.99]	9.46
Jensen 2011	190	604	191	596	0.99 [0.83, 1.17]	11.55
Jeon 2019 (PRODA)	4	30	6	32	0.75 [0.23, 2.42]	0.26
Nobre 2008	5	39	6	40	0.87 [0.29, 2.65]	0.29
Heterogeneity: $\tau^2 = 0.00$, $I^2 = 0.00\%$, $H^2 = 1.00$					0.95 [0.87, 1.04]	
Test of $\theta_i = \theta_j$: Q(6) = 4.44, p = 0.62						
Test of $\theta = 0$: z = -1.01, p = 0.31						
**60-day mortality**						
Bouadma 2010 (PRORATA)	92	311	82	319	1.12 [0.86, 1.45]	5.09
Jensen 2011	231	604	220	596	1.03 [0.88, 1.20]	14.27
Heterogeneity: $\tau^2 = 0.00$, $I^2 = 0.00\%$, $H^2 = 1.00$					1.05 [0.92, 1.20]	
Test of $\theta_i = \theta_j$: Q(1) = 0.29, p = 0.59						
Test of $\theta = 0$: z = 0.70, p = 0.49						
**90-day mortality**						
Dark 2025 (ADAPT-Sepsis)	217	918	215	918	1.01 [0.85, 1.19]	12.28
Bloos 2016	200	552	199	537	0.98 [0.83, 1.16]	12.55
Shehabi 2014	35	200	31	200	1.11 [0.71, 1.74]	1.76
Heterogeneity: $\tau^2 = 0.00$, $I^2 = 0.00\%$, $H^2 = 1.00$					1.00 [0.89, 1.13]	
Test of $\theta_i = \theta_j$: Q(2) = 0.25, p = 0.88						
Test of $\theta = 0$: z = 0.04, p = 0.96						
**Overall**						
Test of group differences: $Q_b(4) = 2.07$, p = 0.72						

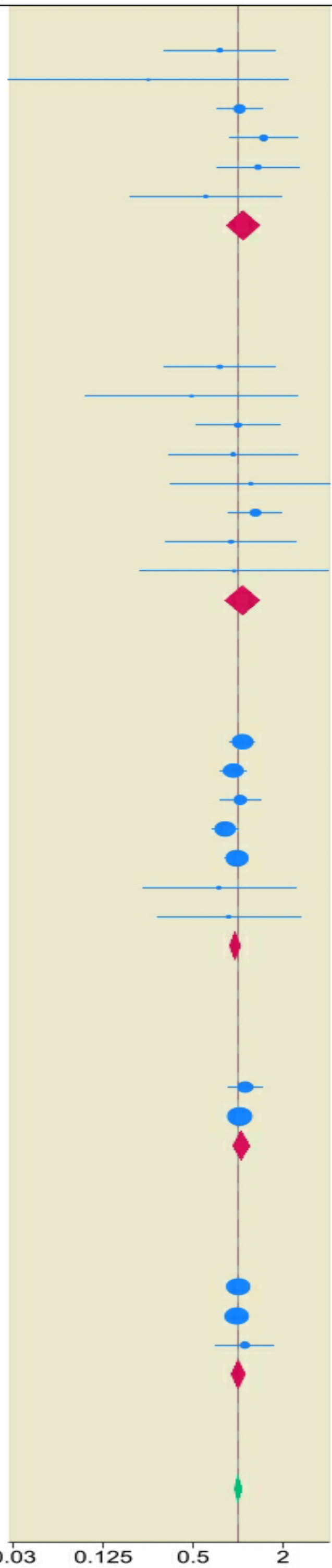

Random-effects DerSimonian–Laird model

Supplementary Figure 3: Subgroup analysis of all-cause mortality according to the mortality time-point

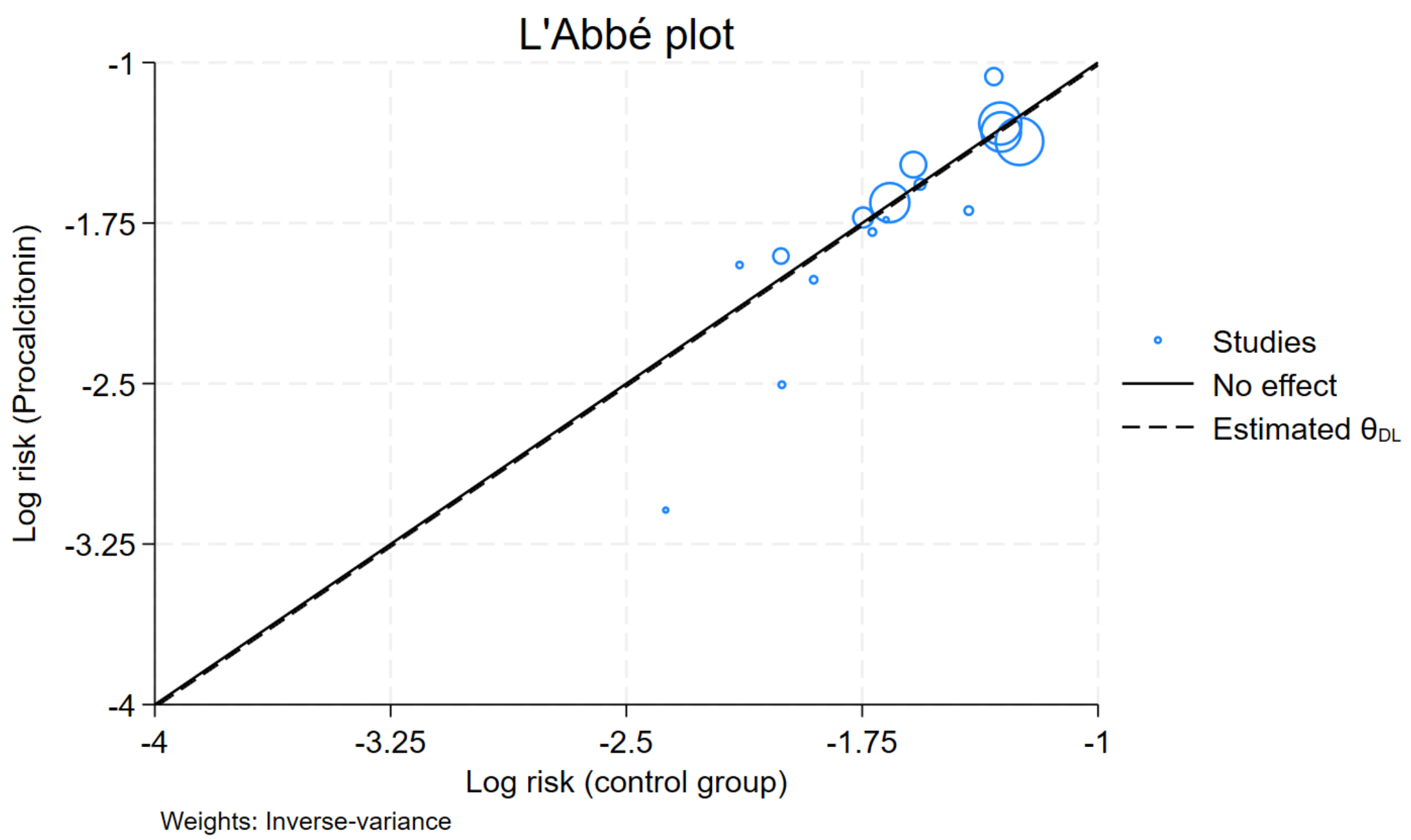

Supplementary Figure 4: L'Abbé plot of all-cause mortality

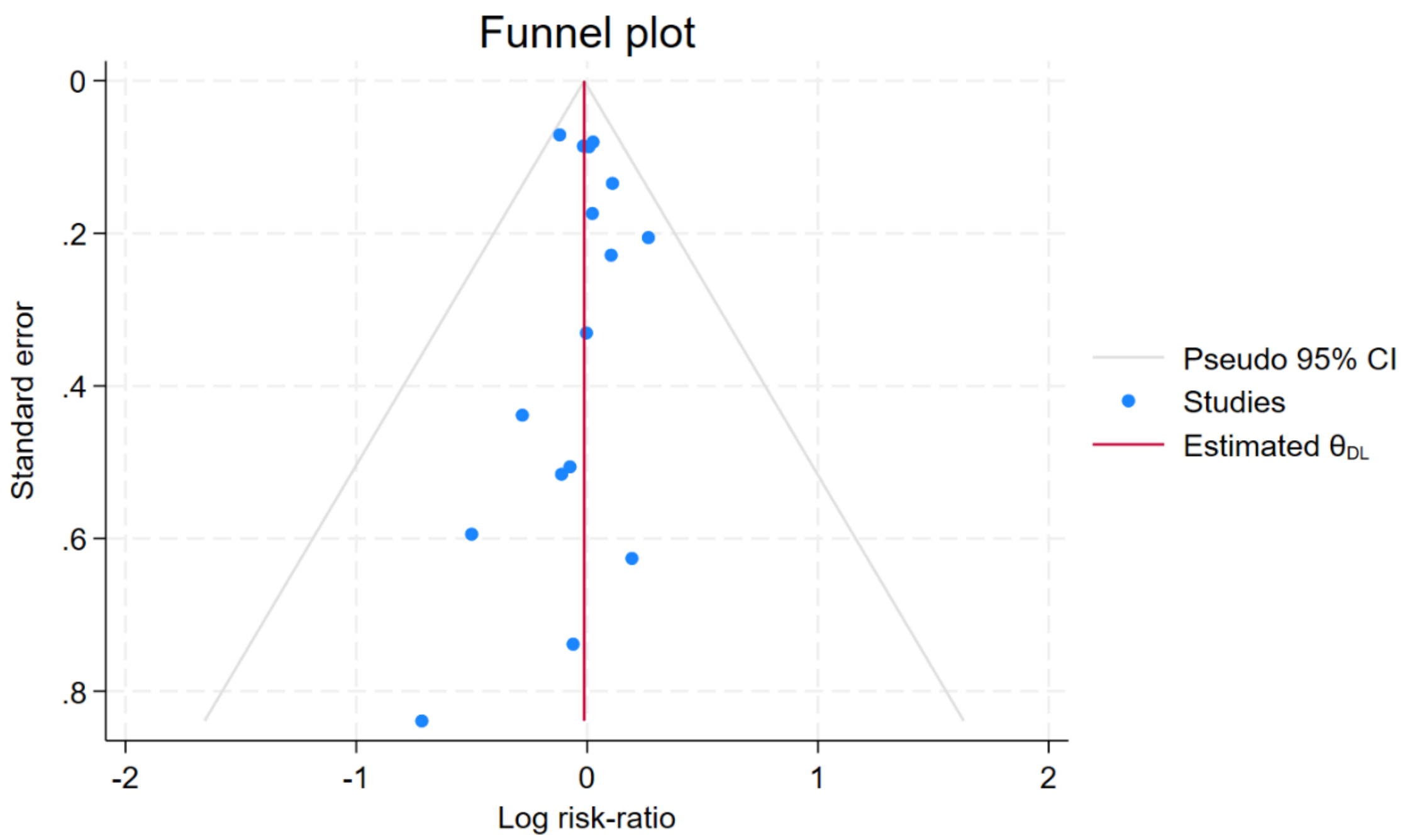

Supplementary Figure 5: Funnel plot of all-cause mortality

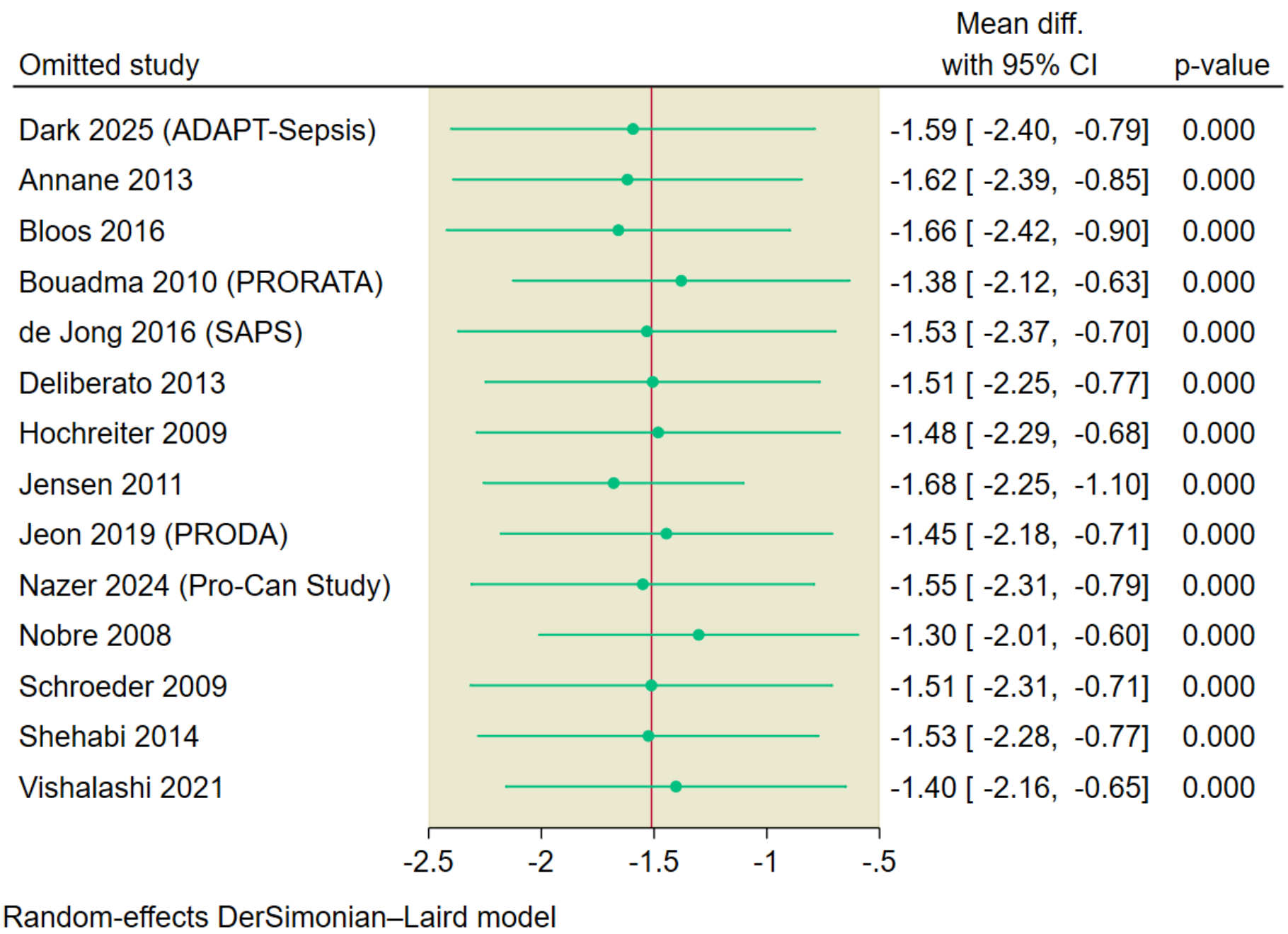

Supplementary Figure 6: Leave one out of the duration of antibiotic therapy

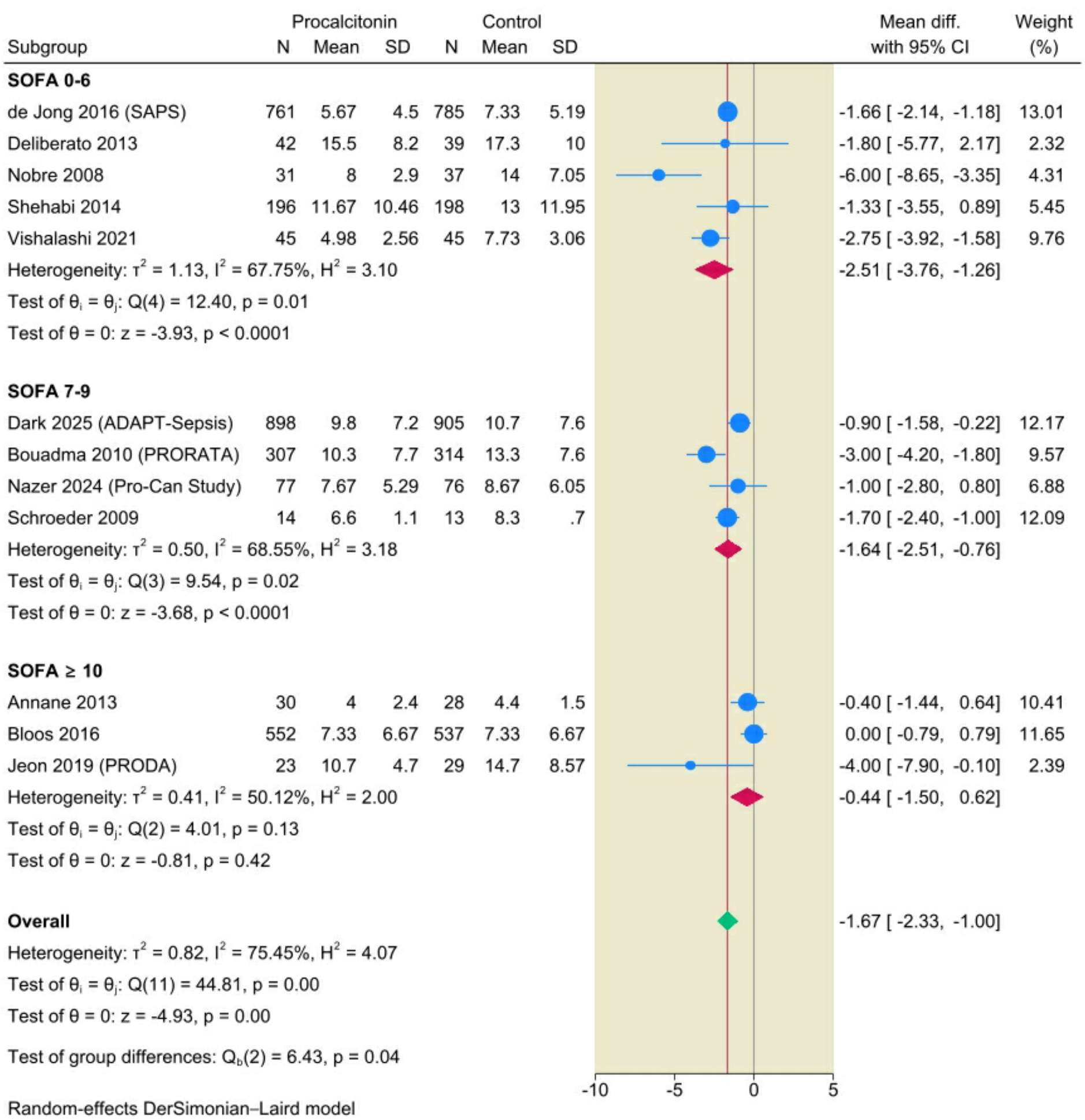

Supplementary Figure 7: Subgroup analyses of duration of antibiotic therapy stratified by baseline SOFA score

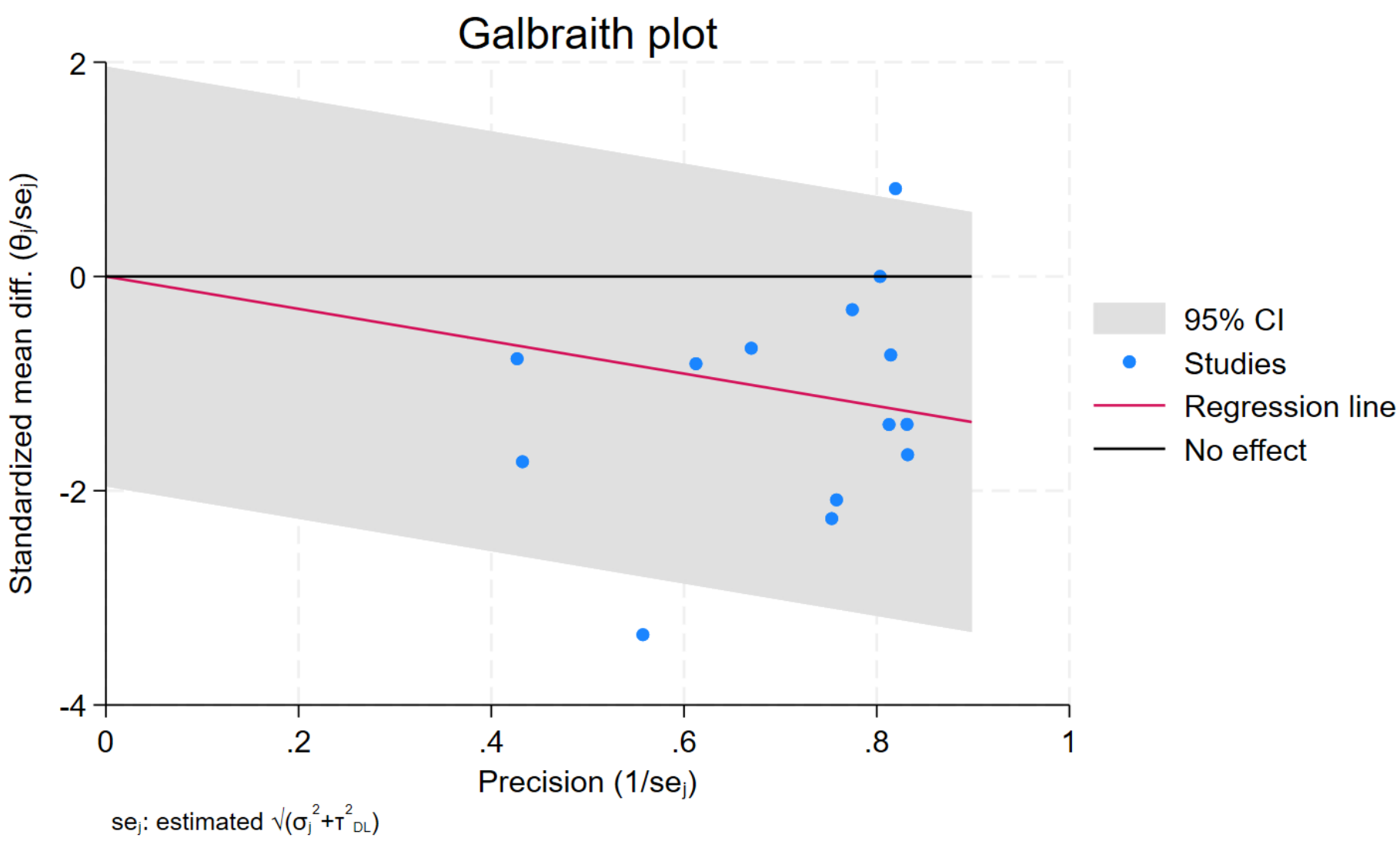

Supplementary Figure 8: Galbraith plot of duration of antibiotic therapy

(A)

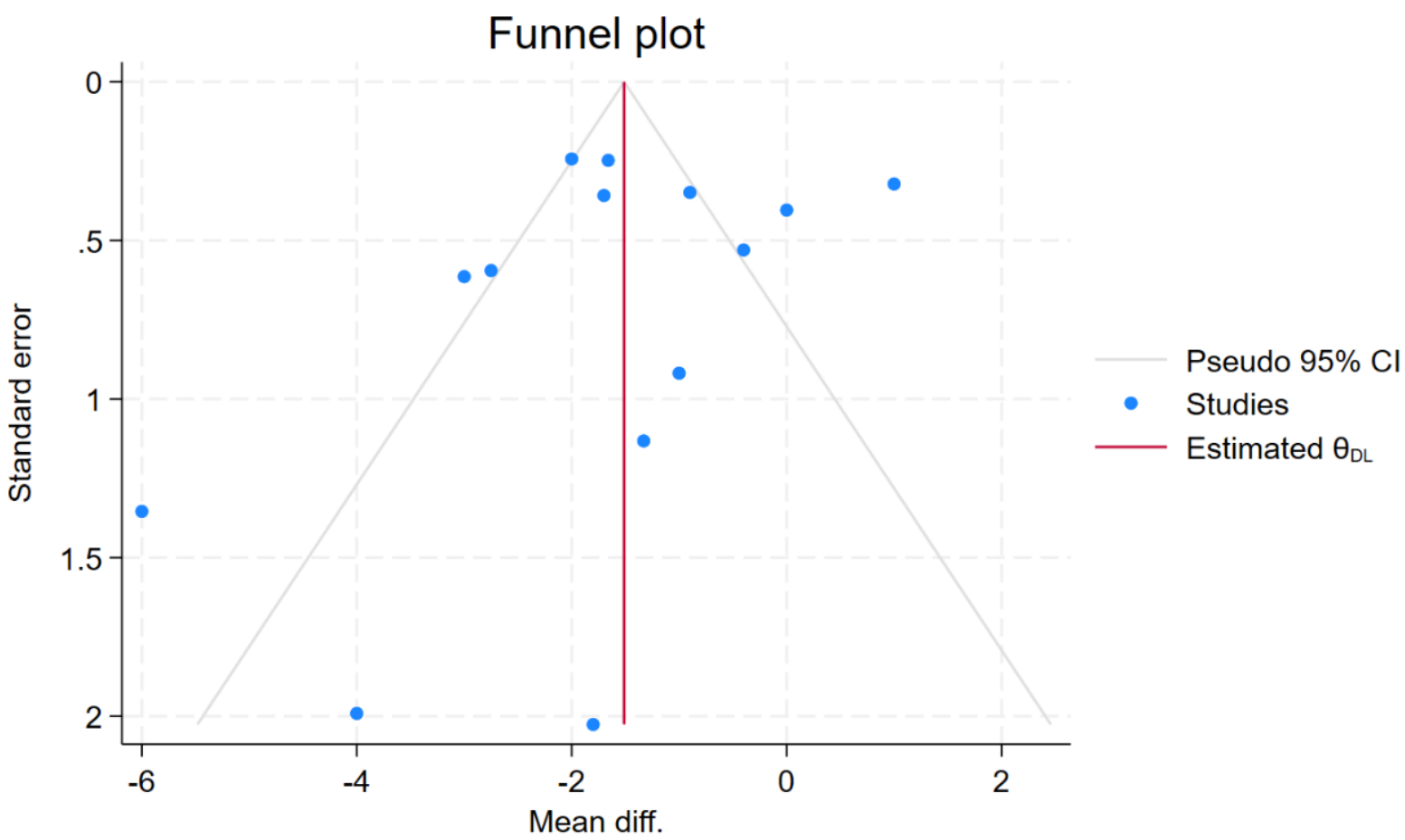

(B)

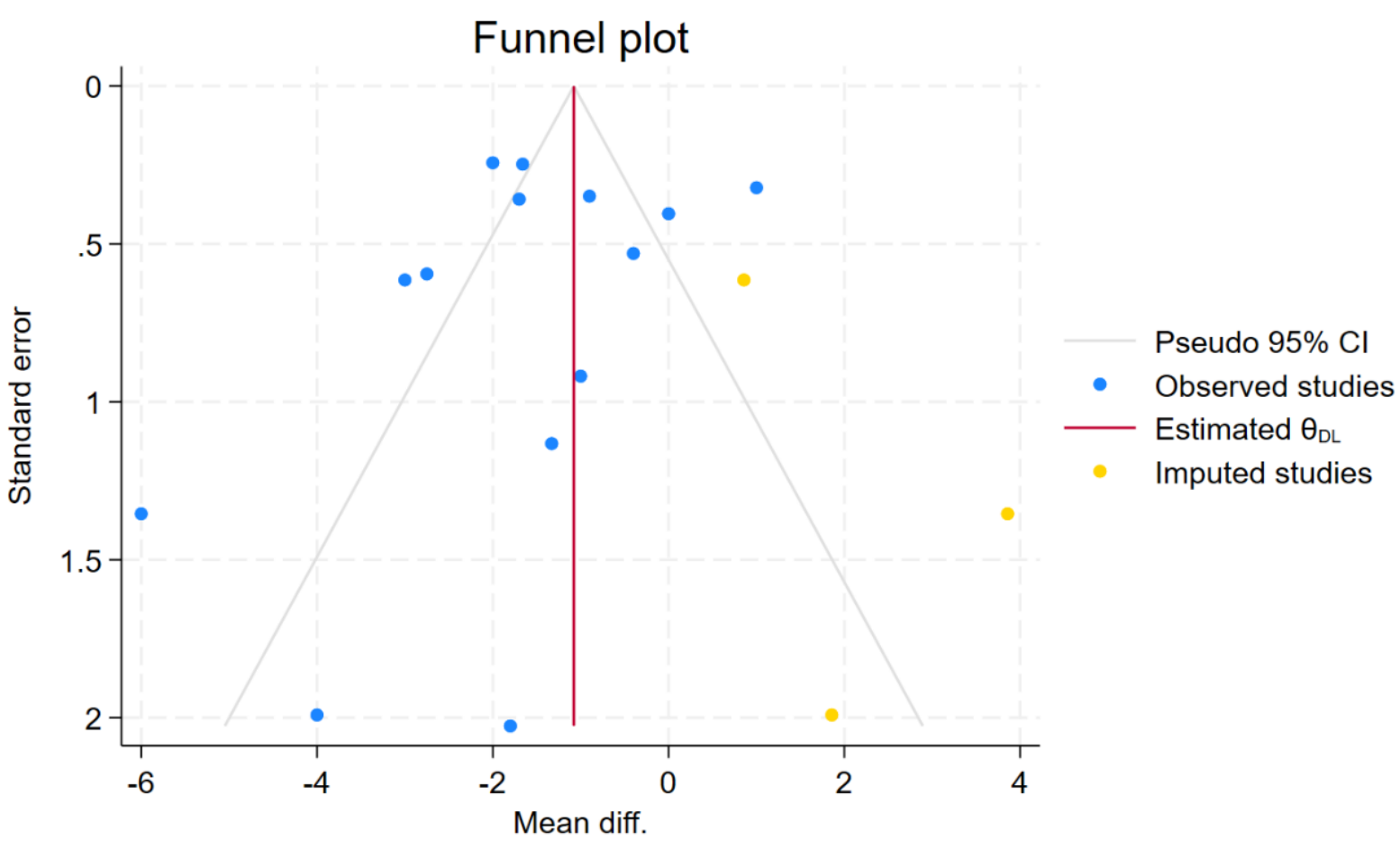

Supplementary Figure 9: Funnel plot for publication bias.

(A) Funnel plot for possible publication bias regarding duration of antibiotic therapy. (B) Funnel plot for possible publication bias using the trim and fill method regarding duration of antibiotic therapy.

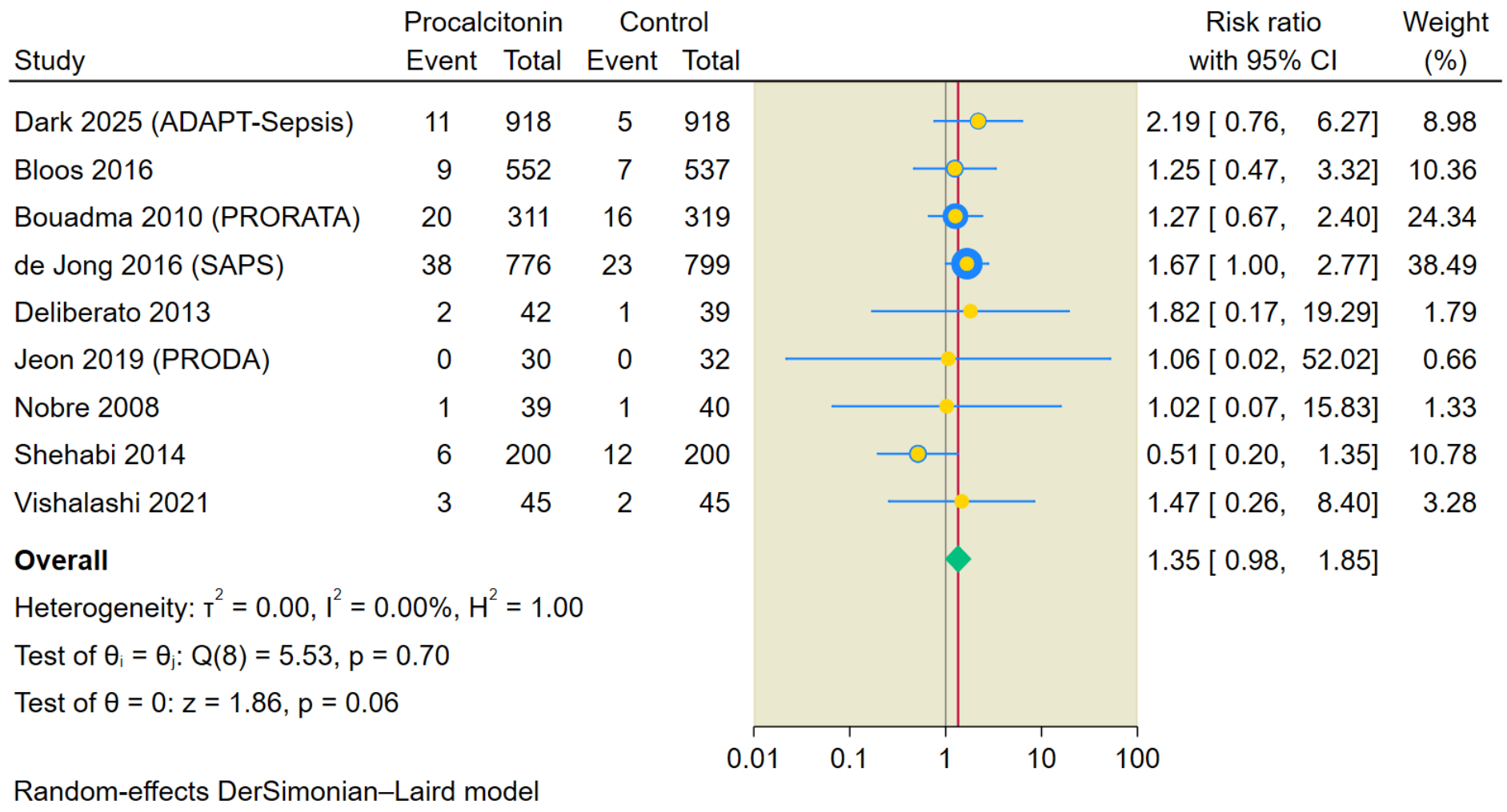

Supplementary Figure 10: Forest plot of infection relapse

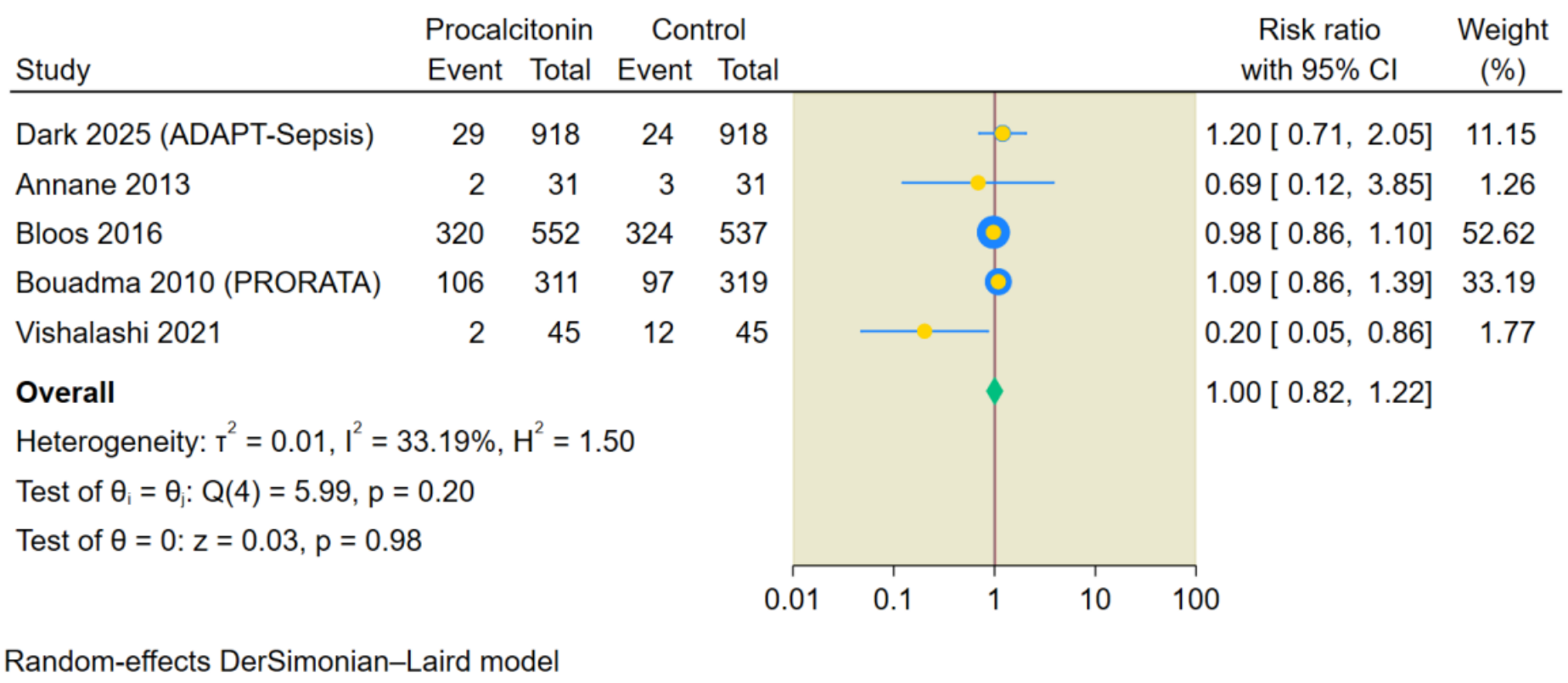

Supplementary Figure 11: Forest plot of new infection or superinfection

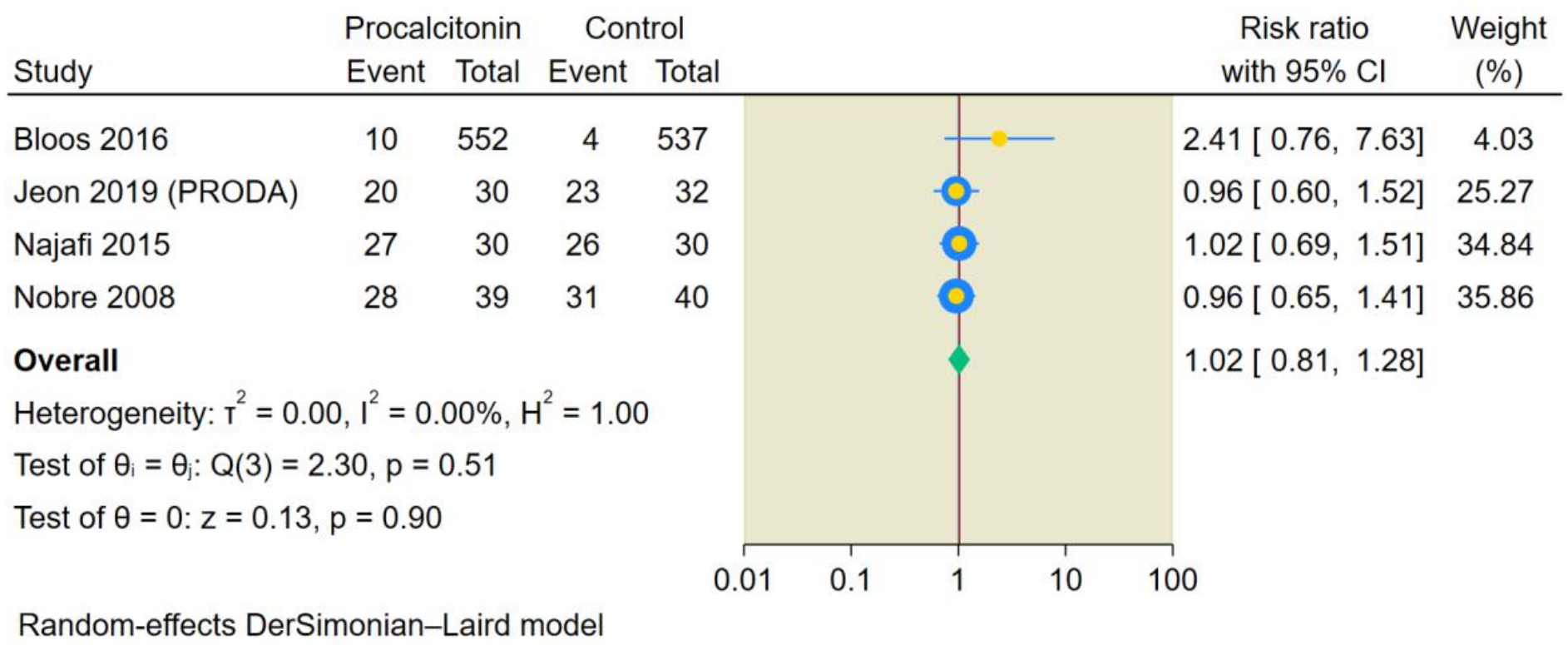

Supplementary Figure 12: Forest plot of clinical cure

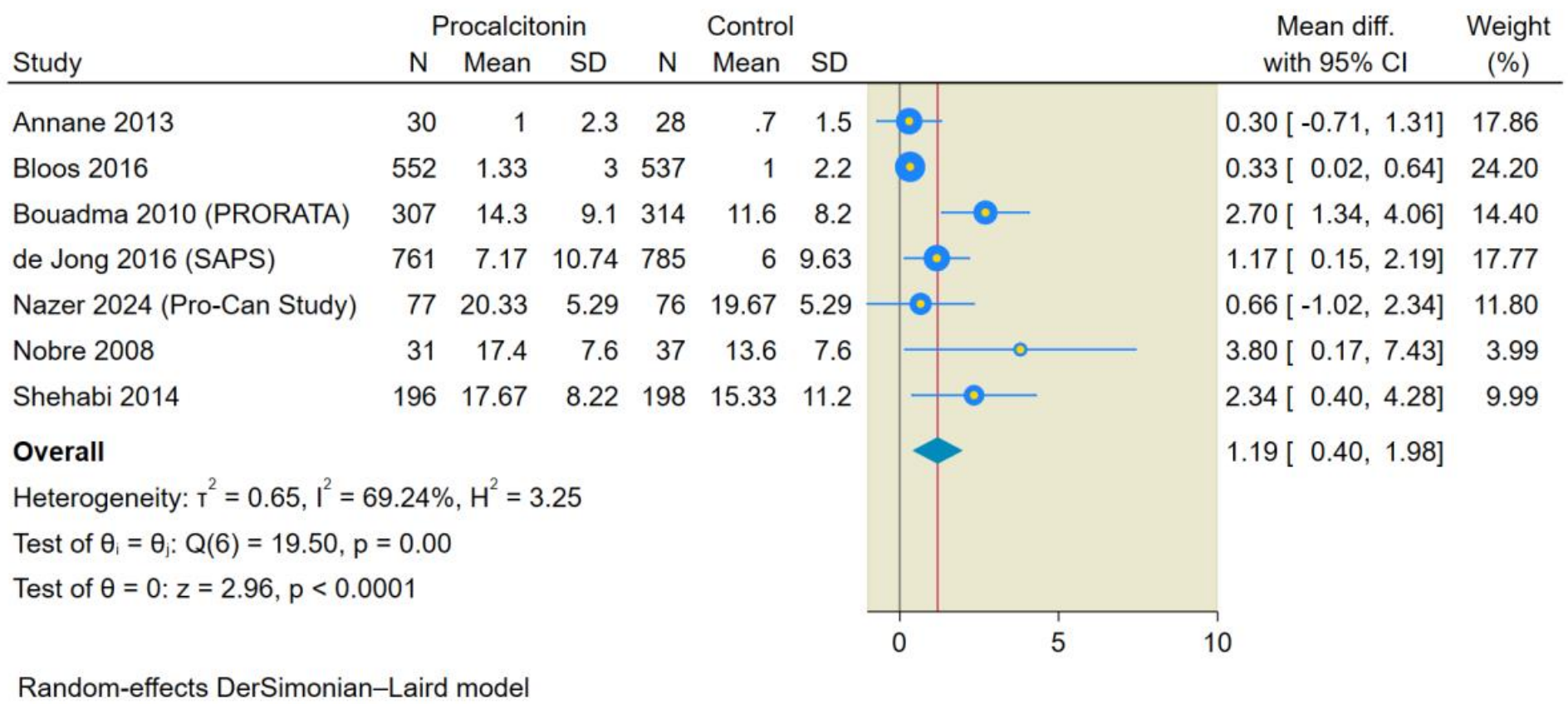

Supplementary Figure 13: Forest plot of antimicrobial-free days

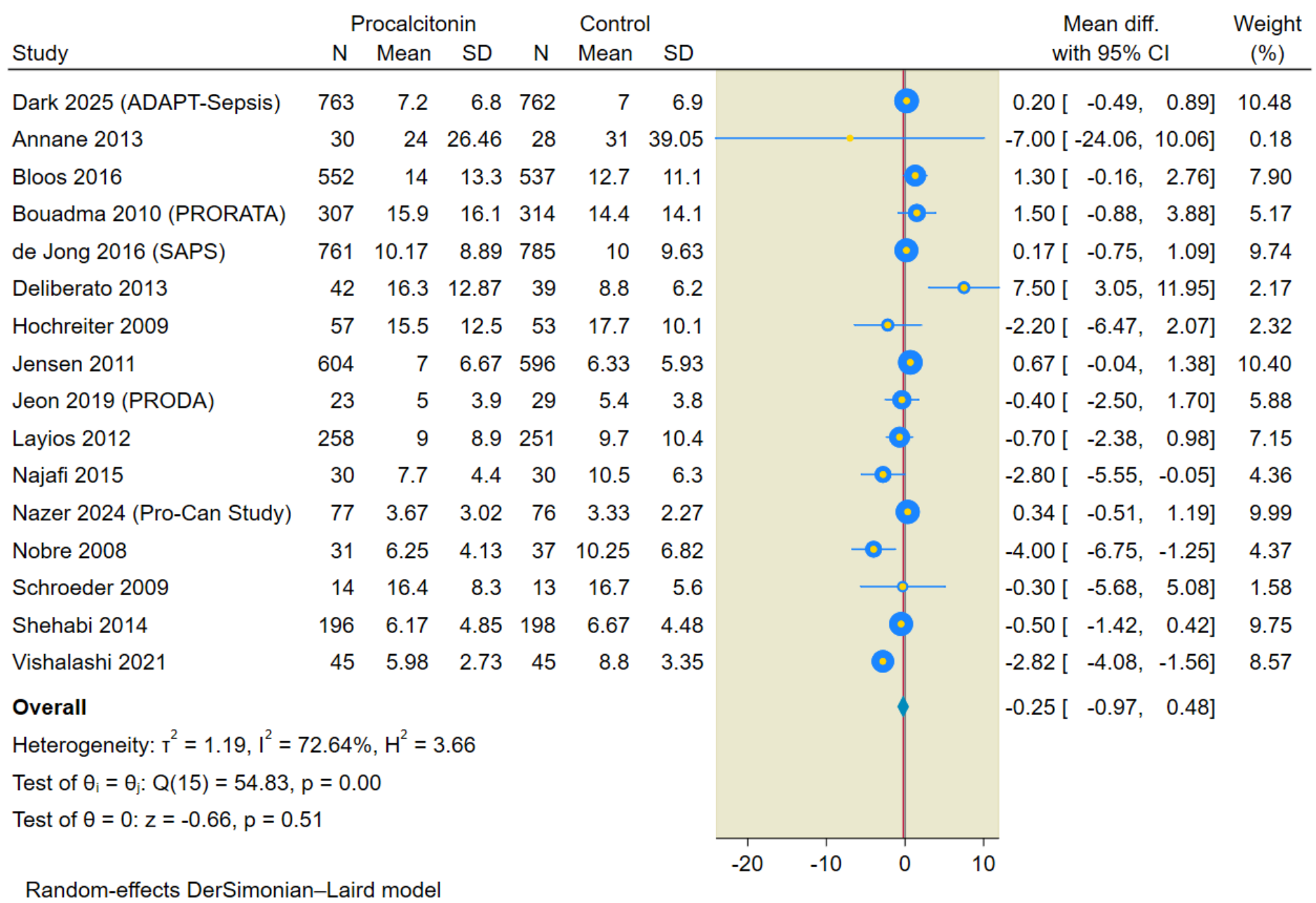

Supplementary Figure 14: Forest plot of length of ICU stay (days)

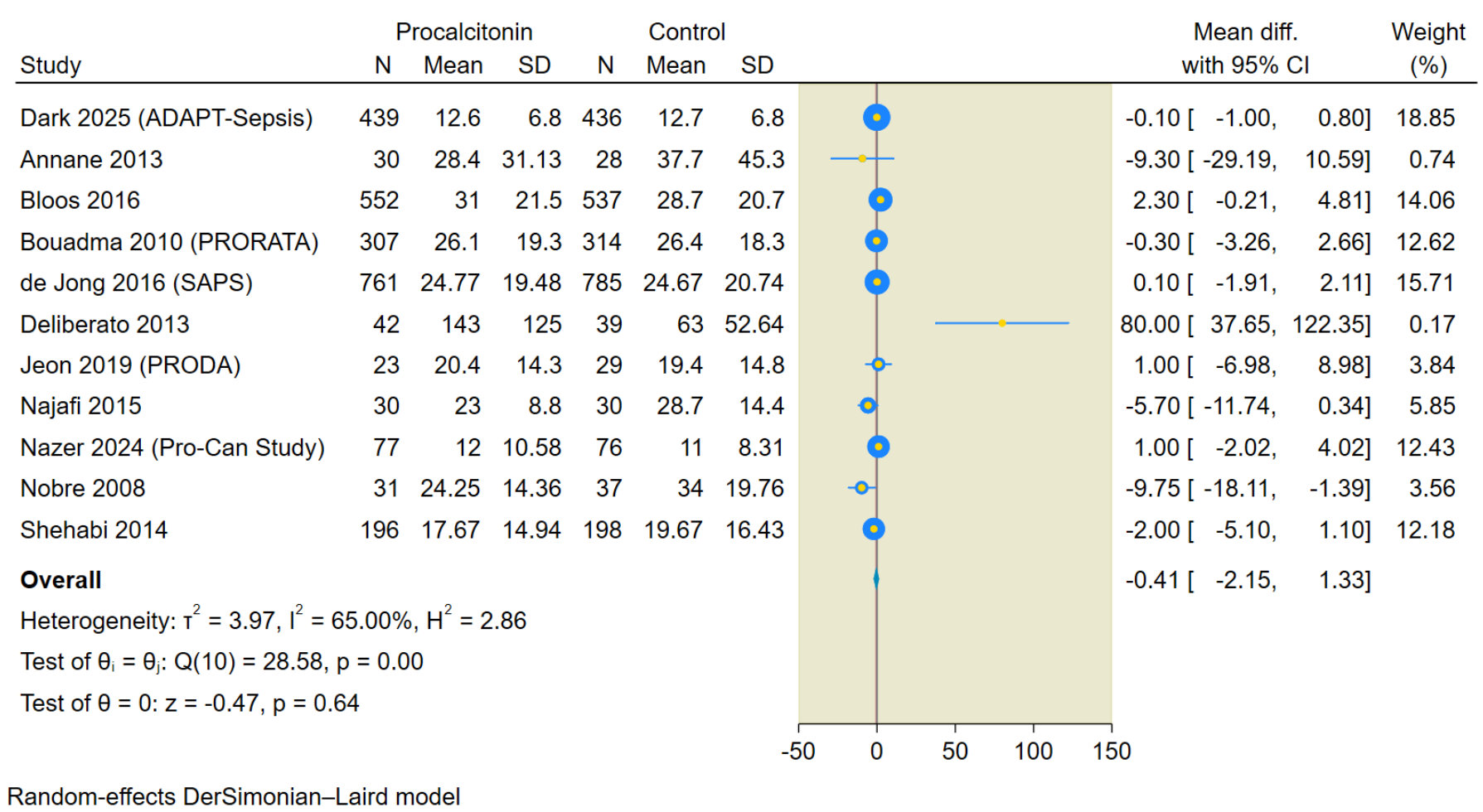

Supplementary Figure 15: Forest plot of length of hospital stay (days)
